# Chemical Proteomics Strategies for Analyzing Protein Lipidation Reveal the Bacterial *O*-Mycoloylome

**DOI:** 10.1021/jacs.4c02278

**Published:** 2024-04-18

**Authors:** Nicholas Banahene, Trenton M. Peters-Clarke, Kyle J. Biegas, Evgenia Shishkova, Elizabeth M. Hart, Amelia C. McKitterick, Nikolas H. Kambitsis, Ulysses G. Johnson, Thomas G. Bernhardt, Joshua J. Coon, Benjamin M. Swarts

**Affiliations:** † Department of Chemistry and Biochemistry, Central Michigan University, Mount Pleasant, Michigan 48859, United States; ‡ Biochemistry, Cell, and Molecular Biology Graduate Programs, Central Michigan University, Mount Pleasant, Michigan 48859, United States; § Department of Chemistry, University of Wisconsin, Madison, Wisconsin 53562, United States; ∥ Department of Biomolecular Chemistry, University of Wisconsin, Madison, Wisconsin 53562, United States; ⊥ National Center for Quantitative Biology of Complex Systems, University of Wisconsin, Madison, Wisconsin 53562, United States; # Department of Microbiology, Harvard Medical School, Boston, Massachusetts 02115 United States; ∇ Howard Hughes Medical Institute, Chevy Chase, Maryland 20815, United States; ○ Morgridge Institute for Research, Madison, Wisconsin 53562, United States

## Abstract

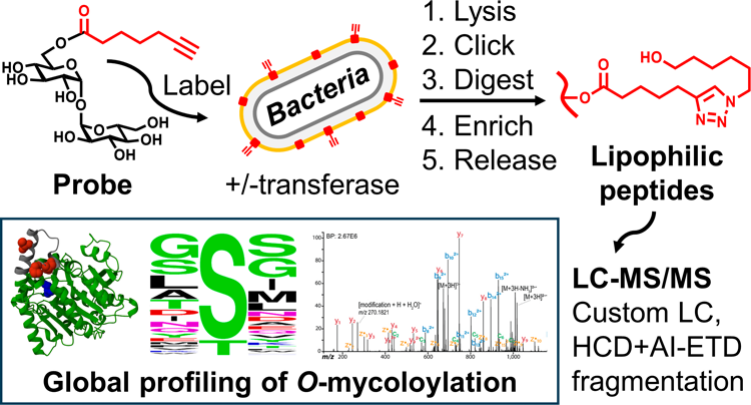

Protein lipidation dynamically controls protein localization and function within cellular membranes. A unique form of protein *O*-fatty acylation in *Corynebacterium*, termed protein *O*-mycoloylation, involves the attachment of mycolic acids—unusually large and hydrophobic fatty acids—to serine residues of proteins in these organisms’ outer mycomembrane. However, as with other forms of protein lipidation, the scope and functional consequences of protein *O*-mycoloylation are challenging to investigate due to the inherent difficulties of enriching and analyzing lipidated peptides. To facilitate the analysis of protein lipidation and enable the comprehensive profiling and site mapping of protein *O*-mycoloylation, we developed a chemical proteomics strategy integrating metabolic labeling, click chemistry, cleavable linkers, and a novel liquid chromatography-tandem mass spectrometry (LC-MS/MS) method employing LC separation and complementary fragmentation methods tailored to the analysis of lipophilic, MS-labile *O*-acylated peptides. Using these tools in the model organism *Corynebacterium glutamicum*, we identified approximately 30 candidate *O*-mycoloylated proteins, including porins, mycoloyltransferases, secreted hydrolases, and other proteins with cell envelope-related functions—consistent with a role for *O*-mycoloylation in targeting proteins to the mycomembrane. Site mapping revealed that many of the proteins contained multiple spatially proximal modification sites, which occurred predominantly at serine residues surrounded by conformationally flexible peptide motifs. Overall, this study (i) discloses the putative protein *O*-mycoloylome for the first time, (ii) yields new insights into the undercharacterized proteome of the mycomembrane, which is a hallmark of important pathogens (e.g., *Corynebacterium diphtheriae*, *Mycobacterium tuberculosis*), and (iii) provides generally applicable chemical strategies for the proteomic analysis of protein lipidation.

## Introduction

**Figure 1:**
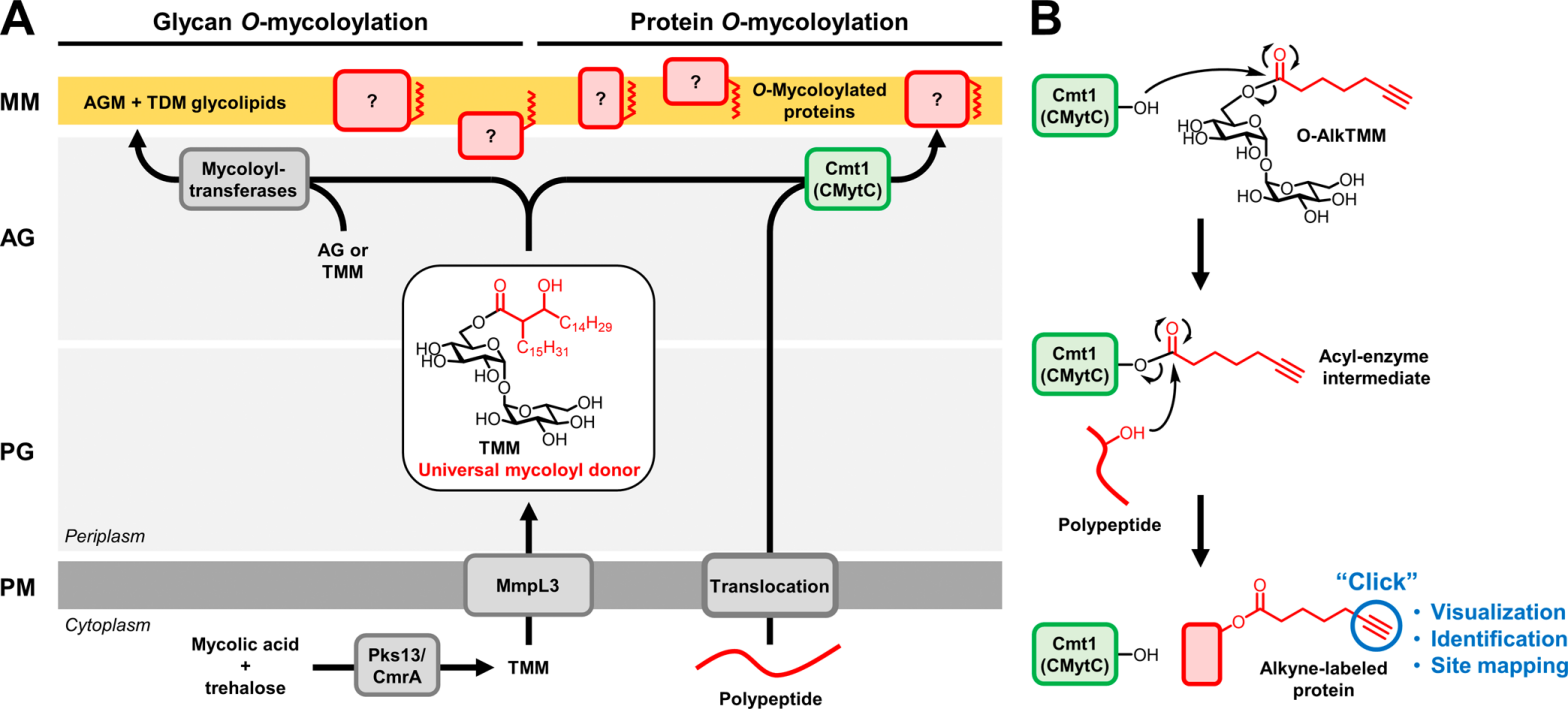
Mycomembrane biosynthesis pathway and exploitation to label and analyze *O*-mycoloylated proteins. (A) Model for the biosynthesis of the major components of the Cg mycomembrane. Cytoplasmic TMM is translocated to the periplasm and used as the universal mycoloyl donor. Subsequently, the TMM donor is processed by mycoloyltransferase enzymes to construct the major components of the mycomembrane, including the glycolipids AGM and TDM (left path) and *O*-mycoloylated proteins, the latter of which are synthesized by the protein-selective mycoloyltransferase Cmt1 (right path). Note: the precise sequence and location of Cmt1-mediated protein *O*-mycoloylation events are not known. (B) Exploitation of mycomembrane biosynthesis to label and analyze *O*-mycoloylated proteins. The synthetic probe O-AlkTMM mimics the mycoloyl donor function of TMM and thus undergoes mycoloyltransferase-mediated alkyne-labeling of the mycomembrane components. In this work, Cmt1-mediated alkyne-labeling of *O*-mycoloylated proteins enabled click chemistry-mediated visualization, identification, and site mapping of this PTM on the whole-proteome scale. AG, arabinogalactan; AGM, arabinogalactan mycolate; MM, mycomembrane; PG, peptidoglycan; PM, plasma membrane; TDM, trehalose dimycolate; and TMM, trehalose monomycolate.

Protein lipidation, a co- or post-translational modification (PTM) that occurs in various forms and in diverse organisms, dynamically regulates the localization, structure, interactions, and functions of proteins.^1,2^ Protein *O*-acylation, the covalent attachment of fatty acids to protein serine or threonine residues through ester bonds, is a relatively uncommon yet functionally critical lipid PTM. For example, Ser-*O*-fatty acylation of two classes of eukaryotic proteins, the Wnt family of proteins and ghrelin, is essential for proper protein localization and biological function.^3,4^ Until recently, examples of protein *O*-acylation were limited to eukaryotic organisms. However, the Daffé and Tropis groups discovered the first example of protein *O*-acylation in bacteria, finding that the cell envelope-associated porins PorA and PorH in *Corynebacterium glutamicum* (Cg), a model organism for pathogens in the *Corynebacterineae* suborder, were *O*-acylated at Ser residues with long-chain branched fatty acids called mycolic acids (Figure 1A).^5^ Furthermore, it was shown that this lipidation, termed protein *O*-mycoloylation, is essential for the localization of porins to Cg’s mycolic acid-rich outer membrane, or “mycomembrane”, as well as for their channel activity, suggesting that *O*-mycoloylation may play a role in dynamically targeting proteins to the mycomembrane.^5−7^ The lipid transferase that catalyzes protein *O*-mycoloylation in Cg, Cmt1 (also referred to as CMytC^8^), is necessary for normal bacterial growth and antibiotic tolerance, pointing to the physiological significance of this PTM.^9^ More broadly, mycolic acids, mainly in the form of the glycolipids arabinogalactan mycolate (AGM) and trehalose dimycolate (TDM), are the major components of the indispensable, protective mycomembrane of hundreds of related species in the *Corynebacterineae* suborder.^10−12^ In addition to Cg, this suborder of bacteria also includes the pathogens *Corynebacterium diphtheriae*, *Mycobacterium tuberculosis*, *Mycobacterium leprae*, and others, which collectively present a massive global health burden.^13^

Despite intense interest in characterizing the proteomic composition of the mycomembrane toward the discovery of novel drug targets,^14−16^ the extent to which protein *O*-mycoloylation occurs beyond a few mycomembrane porins in Cg is unknown, and there remains little information about the roles of *O*-mycoloylation in protein structure, localization, and function. These knowledge gaps underscore the limited ability of traditional methods to analyze protein–lipid PTMs on the whole-proteome scale.^17^ As with many other protein PTMs, the global analysis of protein lipidation using conventional bottom-up liquid chromatography-tandem mass spectrometry (LC-MS/MS) proteomics is inherently difficult due to the dynamic nature of the modification, the difficulty of detecting low-abundance-modified peptides, and the presence of confounding corresponding unmodified peptides. In addition, fatty acyl groups—and, in particular, *O*-mycoloyl groups—are often large, hydrophobic, and neutral and may be attached to peptides through relatively labile bonds (e.g., esters and thioesters), all of which can complicate the analysis of lipidated peptides by traditional LC-MS/MS methods. Moreover, bioinformatic methods for predicting protein lipidation may not be suitable for lipid modifications like *O*-mycoloylation that are newly discovered, are not well-characterized, or do not have a defined consensus sequence. Therefore, several properties intrinsic to lipid PTMs make them difficult to investigate, and these problems are amplified in the context of protein *O*-mycoloylation.

Recent advances in metabolic labeling, click chemistry, and mass spectrometry have converged to enable the development of powerful chemical proteomics strategies for the analysis of PTMs.^18−20^ Typically, in these approaches, a chemical probe is metabolically incorporated into target proteins in live cells, thus installing a chemical handle (e.g., alkyne or azide) onto the PTM. The installed handle can then be click-conjugated to functional tags that permit specific visualization and enrichment, followed by trypsin digestion and LC-MS/MS analysis. Such methods have been developed for the analysis of various types of protein lipidation, including different forms of fatty acylation, prenylation, cholesterylation, and glycosylphosphatidylinositol anchorage.^2,17,21−24^ With respect to bacterial protein *O*-mycoloylation, recent work by us^25^ and Bayan^26^ used metabolic labeling with chemical probes to visualize *O*-mycoloylated proteins by SDS-PAGE and Western blot. However, both of these prior studies on *O*-mycoloylated proteins were limited to a handful of porin proteins that were either previously known or suspected to be *O*-mycoloylated. Thus, to date, there has been no comprehensive, unbiased study of *O*-mycoloylation on the whole-proteome scale, precluding a more holistic understanding of the scope, nature, and functional consequences of this PTM.

In this study, we harnessed chemical proteomics to accomplish the first elucidation of the bacterial protein *O*-mycoloylome. We established a method for specific metabolic labeling of *O*-mycoloylated proteins in live bacterial cells, in which a chemical probe designed to mimic the mycoloyl donor substrate is processed by the lipid transferase Cmt1 to install alkynes onto proteins (Figure 1B). We then used click chemistry to label alkyne-modified proteins with fluorophore and/or affinity tags, including variants with cleavable linkers, which allowed protein/peptide visualization, pull-down, and global profiling and site mapping using a novel LC-MS/MS method that was optimized for the separation and detection of lipophilic, MS-labile modified peptides. Our results revealed that *O*-mycoloylation is significantly more widespread than previously known, as we identified ∼30 proteins with high confidence, many with ≥2 predicted *O*-mycoloylation sites occurring at serine and, less commonly, threonine residues. In addition to porins, our data demonstrate Cmt1-dependent lipid modification of numerous proteins with known functions in cell envelope synthesis, remodeling, or transport, including multiple mycoloyltransferases. Many of the proteins identified were previously predicted to be mycomembrane-associated, which further supports the hypothesis that *O*-mycoloylation targets proteins to the mycomembrane. Given that the mycomembrane is a prime target for diagnostic and therapeutic development, but its proteome remains poorly characterized, this study motivates extended studies of protein *O*-mycoloylation in related organisms of medical significance. More broadly, this study represents the first comprehensive characterization of a protein *O*-acylation PTM of bacterial origin and discloses new strategies that may be generally applicable to the investigation of protein lipidation.

## Results and Discussion

### Design of Chemical Proteomics Strategies to Label and Analyze *O*-Mycoloylated Proteins

To select a strategy for the metabolic labeling of *O*-mycoloylated proteins, we considered the relevant biosynthetic pathway depicted in Figure 1A. The biosynthesis of AGM, TDM, and *O*-mycoloylated proteins proceeds via a shared general pathway that is conserved across the *Corynebacterineae* suborder.^12,27^ In the cytoplasm, two activated fatty acid precursors are joined to generate mycolic acid, which is subsequently linked to the disaccharide trehalose to form trehalose monomycolate (TMM). TMM is then flipped to the periplasm, where it serves as the universal mycoloyl donor to build the mycomembrane. The 6-*O*-mycoloyl group of the TMM donor is transferred onto various acceptor molecules by mycoloyltransferases, a family of secreted lipid transferase enzymes.^28^ The acceptor molecules receiving mycoloyl groups from the TMM donor include arabinogalactan to form AGM, another molecule of TMM to form TDM, and, at least in Cg, polypeptide serine residues to form *O*-mycoloylated proteins. Among the six mycoloyltransferase isoforms encoded by the Cg genome, which mainly function in AGM and TDM biosynthesis,^29^ the Cmt1 isoform was found to be exclusively responsible for *O*-mycoloylation of polypeptide acceptors, including the porins PorA and PorH.^30^

In view of this biosynthetic pathway, we considered two possible ways to metabolically label *O*-mycoloylated proteins: TMM- or fatty acid-based probes. Our lab previously introduced TMM-based probes that mimic the mycoloyl donor function of TMM. We demonstrated that synthetic TMM analogues containing truncated 6-*O*-acyl chains with various chemical tags (e.g., alkyne, azide, fluorophore) undergo mycoloyltransferase-mediated transfer of their acyl groups onto acceptor glycans—thus generating labeled AGM and TDM—in live cells of Cg and related mycobacterial species.^31,32^ On the basis that TMM is the universal mycoloyl donor, we hypothesized that TMM-based probes would also label *O*-mycoloylated proteins, as schematized in Figure 1B. Consistent with this hypothesis, in a prior proof-of-concept study, we found that O-AlkTMM, a TMM analogue containing a clickable acyl chain with a terminal alkyne, indeed labeled *O*-mycoloylated porin proteins in Cg.^25^ This study, which focused on a chloroform–methanol extract of Cg containing a few small hydrophobic proteins, confirmed that PorA and PorH were modified with mycoloyl groups and also suggested that the anion-selective channel protein PorB is also modified.^25^ In addition to TMM probes, fatty acid probes have been investigated for labeling *O*-mycoloylated proteins. The Bayan group used a targeted protein overexpression system in Cg in combination with a fatty acid-based probe, 17-octadecynoic acid, to confirm *O*-mycoloylation of PorB and the related porin PorC.^26^ Thus, in these limited-scope studies, both TMM- and fatty acid-based probes were shown to label *O*-mycoloylated porins. In considering which labeling approach to adopt in the present proteomic study, we favored the use of TMM probes for several reasons. TMM probes (i) contain the trehalose motif recognized by mycoloyltransferases, which confers specificity; (ii) intercept the protein *O*-mycoloylation pathway at a “late” stage, which promotes efficient incorporation and suppresses off-target labeling; and (iii) are designed to incorporate via an extracellular route, which should avoid the need for the probe to cross the entire cell envelope. In addition, an adjustable design feature of TMM probes is their 6-*O*-acyl chain, which can be significantly simplified relative to native TMM and still undergo incorporation into mycoloyl acceptor molecules due to the high substrate tolerance of mycoloyltransferases.^31,32^ This flexibility allowed us to select a TMM probe that would deposit a relatively small acyl group onto proteins, which we anticipated would facilitate the downstream LC-MS/MS detection of lipidated peptides. Based on the above considerations, we chose O-AlkTMM as the metabolic labeling probe for this study.

**Figure 2:**
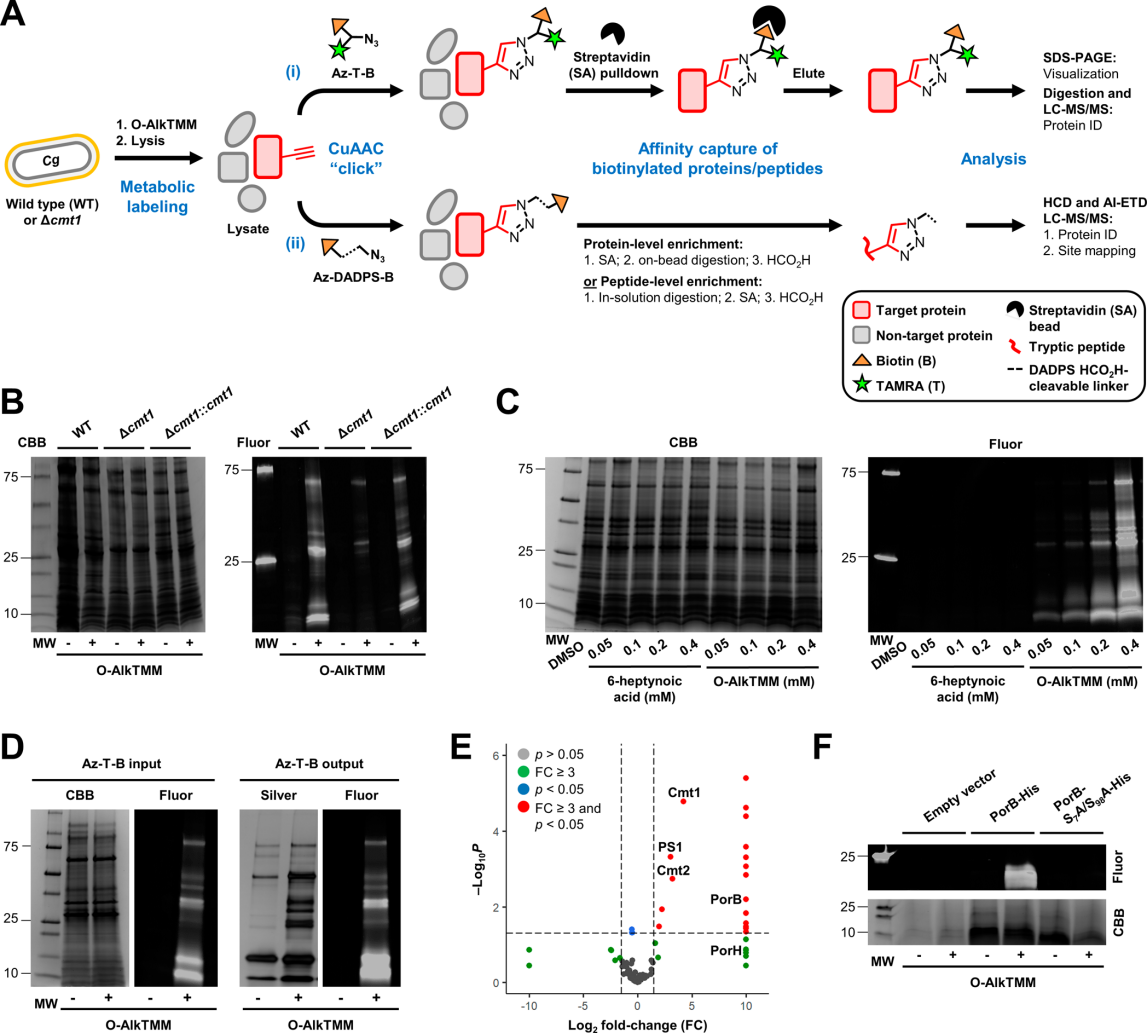
Specific labeling and identification of putative *O*-mycoloylated proteins. (A) Chemical proteomics strategies developed in this study to enable the visualization, identification, and site mapping of *O*-mycoloylated proteins. (B) Cg wild type (WT), *cmt1* mutant (Δ*cmt1*), or complement (Δ*cmt1*::*cmt1*) were treated with O-AlkTMM or left untreated, then cell lysates were collected and subjected to CuAAC with azido-488 and analyzed by SDS-PAGE with visualization by Coomassie Brilliant Blue (CBB) staining and in-gel fluorescence scanning (Fluor). (C) Cg WT was treated with varying concentrations of O-AlkTMM, 6-heptynoic acid, or left untreated and then processed and analyzed as in (B). (D) As depicted in (Ai), lysates from O-AlkTMM-treated Cg WT were subjected to CuAAC with Az-T-B, and then the fluorescently labeled, biotinylated proteins (“input”) were enriched on streptavidin-coated beads and eluted (“output”). Input and output samples were analyzed by SDS-PAGE with visualization by CBB staining or silver staining, respectively, and in-gel fluorescence scanning. (E) Four replicate output samples prepared as in (D) were digested, and the resulting peptides were analyzed by label-free quantitative LC-MS/MS. The volcano plot depicts proteins in red that were identified as significantly enriched (*p* < 0.05) by a fold-change (FC) of at least 3 in the O-AlkTMM-treated samples versus the untreated controls. Proteins of interest are annotated and discussed in the main text. Raw and curated data from this study (LC-MS/MS study 1 (Az-T-B)) are found in Supporting Tables S4–S6. (F) Cg strains expressing either His-tagged wild-type PorB, His-tagged double mutant PorB-S_7_A/S_98_A, or empty vector control were treated with O-AlkTMM or left untreated; then, cell lysates were collected and subjected to CuAAC with azido-488. His-tagged proteins were enriched on Ni^2+^-NTA resin, eluted, and analyzed by SDS-PAGE with visualization by CBB staining and in-gel fluorescence scanning.

We envisioned that O-AlkTMM-based labeling could be used in combination with click chemistry and LC-MS/MS workflows to enable the specific visualization, identification, and PTM site mapping of *O*-mycoloylated proteins on the whole-proteome level (Figure 2A). To permit protein visualization or identification by conventional chemical proteomics, we utilized the strategy shown in Figure 2Ai. In this scenario, alkyne-labeled protein lysates from Cg would be subjected to a Cu-catalyzed azide–alkyne cycloaddition (CuAAC) reaction with azido–TAMRA–biotin (Az-T-B), thereby installing both fluorophore and affinity handles onto target proteins. Subsequently, fluorescently tagged and biotinylated proteins would be affinity-enriched on streptavidin beads and then eluted and either (i) visualized by SDS-PAGE with fluorescence scanning or (ii) identified by proteolytic digestion and standard LC-MS/MS proteomic analysis. In a parallel and complementary strategy to identify proteins and, importantly, elucidate the sites of *O*-mycoloylation, we designed the lipid-centric strategy shown in Figure 2Aii. In this scenario, alkyne-labeled proteins would be subjected to CuAAC reaction with an azido–biotin reagent containing a formic acid (HCO_2_H)-cleavable dialkoxydiphenylsilane linker (Az-DADPS-B).^33^ The use of cleavable linkers enables efficient protein- or peptide-level enrichment of modified peptides bearing small MS-detectable chemical handles, which aids in PTM site mapping.^18,34^ Following enrichment of modified peptides, we used a custom LC-MS/MS method optimized to separate and detect modified peptides using two complementary fragmentation methods, higher-energy C-trap dissociation (HCD) and activated ion electron-transfer dissociation (AI-ETD). This LC-MS/MS approach was designed to maximize sequence coverage and localization of modification sites for hydrophobic peptides with relatively labile *O*-acyl modifications. Together, the complementary chemical proteomics strategies depicted in Figure 2A were designed to provide (i) an unprecedented level of detail regarding the scope and nature of bacterial protein *O*-mycoloylation and (ii) generally applicable chemical proteomics strategies specifically designed to facilitate the analysis of lipid PTMs.

### Specific Labeling and Visualization of *O*-Mycoloylated Proteins

Our designed strategies rely on the ability of O-AlkTMM to efficiently label *O*-mycoloylated proteins with high specificity. As noted above, in prior work, we demonstrated that O-AlkTMM labeled low-molecular-weight *O*-mycoloylated porins present in a Cg chloroform–methanol extract containing a small number of total proteins (∼6 proteins were observed in this extract by SDS-PAGE analysis with Coomassie staining).^25^ In the present study, we began by investigating the scope and specificity of O-AlkTMM-mediated protein labeling in lysates from Cg. Based on the prior finding that Cmt1 is the only known enzyme responsible for transferring mycoloyl groups from the donor TMM onto polypeptide substrates in Cg,^30^ we constructed a *cmt1* deletion mutant (Δ*cmt1*) and its corresponding complement (Δ*cmt1*::*cmt1*) in Cg wild-type strain MB001, which were used to aid in the evaluation of probe specificity (see Supporting Tables S1–S3 for lists of strains, plasmids, and primers used in this study). After the treatment of WT and mutant Cg strains with O-AlkTMM, cell lysates were collected using a procedure optimized to efficiently extract labeled proteins (Supporting Figure S1)^35^ and then subjected to CuAAC reaction with azido-488 fluorophore, resolved by SDS-PAGE, and analyzed by Coomassie staining and in-gel fluorescence scanning. Whereas O-AlkTMM efficiently labeled proteins of varied sizes in WT Cg, very little labeling was observed in the Cmt1-deficient mutant (Δ*cmt1*), and labeling was fully restored in the complement (Δ*cmt1*::*cmt1*) (Figure 2B). Two faint bands in the Δ*cmt1* mutant lysates were observed at ∼35 and 70 kDa, which represented a minor amount of Cmt1-independent labeling that could be due to labeling via other mycoloyltransferase isoforms or nonspecific incorporation of the probe (discussed further below in the context of PTM site mapping data). We also found that O-AlkTMM labeled WT Cg proteins in a concentration-dependent manner and that a trehalose-deficient control compound, 6-heptynoic acid, failed to label proteins, further supporting the specific metabolic incorporation of O-AlkTMM (Figure 2C).

Additionally, we compared labeling by O-AlkTMM to the alkyne fatty acid probe 17-octadecynoic acid, which was previously reported to label PorB and PorC using a targeted protein overexpression system.^26^ Although 17-octadecynoic acid efficiently incorporated into Cg cells and proteins, labeling of higher-molecular-weight proteins was less efficient and numerous proteins in the 25–75 kDa range were labeled in the Δ*cmt1* mutant, implying substantial off-target labeling (Supporting Figure S2). This was not unanticipated, given that alkyne fatty acids have previously been shown to label proteins in a variety of Gram-negative, Gram-positive, and mycobacterial species.^36^ Overall, our data demonstrate that O-AlkTMM, by virtue of mimicking the universal mycoloyl donor TMM, efficiently labels Cg proteins in a Cmt1-dependent manner and thus accurately reports on protein *O*-mycoloylation. Furthermore, numerous proteins in addition to the previously identified low-molecular-weight porins were labeled by O-AlkTMM, suggesting that protein *O*-mycoloylation occurs more broadly than previously known.

### Proteome-Wide Enrichment and Identification of *O*-Mycoloylated Proteins

With confidence that O-AlkTMM specifically labels *O*-mycoloylated proteins, we next used the probe to affinity-enrich and identify labeled proteins via LC-MS/MS using the strategy shown in Figure 2Ai. O-AlkTMM-labeled lysates from Cg were subjected to CuAAC with Az-T-B; then, the fluorescently labeled and biotinylated protein products (“input” samples) were captured on streptavidin-coated beads, washed, and released by boiling (“output” samples). SDS-PAGE analysis of input and output samples, aided by the attached TAMRA fluorophore, confirmed that the O-AlkTMM-labeled proteins were successfully enriched using this method (Figure 2D). Next, enriched proteins from output samples were trypsin-digested, and the resulting peptide samples were analyzed by label-free quantitative LC-MS/MS employing HCD fragmentation. This LC-MS/MS study is referred to herein as “LC-MS/MS study 1 (Az-T-B)”. Out of 168 individual proteins identified across all replicates, 21 proteins were identified as enriched by at least 3-fold in probe-treated versus untreated samples with statistical significance (*p* < 0.05) (Figure 2E; all additional raw and curated data for all LC-MS/MS studies in this work can be found in Supporting Tables S4–S11; LC-MS/MS data for study 1 are located in Supporting Tables S4–S6). Out of these 21 identified proteins, 15 (75%) were exclusively identified in all probe-treated replicates and were absent from the negative control replicates. Furthermore, of the 21 proteins, approximately half (43%) were predicted in a prior study by Marchand et al. as possible mycomembrane-associated proteins based on cellular fractionation and proteomic analysis (Supporting Figure S3A and Table S6),^37^ which is an additional indicator of the specificity of the labeling strategy and implicates this PTM in targeting proteins to the mycomembrane.

Promisingly, included among the proteins exclusively identified in probe-treated samples were PorB and PorH, both of which are mycomembrane-associated porins that are known to be *O*-mycoloylated.^5,25,26^ Although PorH failed to meet our inclusion criteria because it was identified in only two replicates, likely owing to the inherent difficulty of detecting small proteins in bottom-up LC-MS/MS proteomics,^38^ its exclusive detection in the probe-treated sample and its previously reported labeling by *O*-AlkTMM^25^ suggest that this is indeed an authentic modification. The finding that 2 out of the ∼20 identified proteins are among the few proteins previously known to be *O*-mycoloylated, in an organism that expresses >1000 proteins detectable by LC-MS/MS,^39^ is an additional convincing validation that the O-AlkTMM labeling strategy is on target. In addition to these previously known *O*-mycoloylated porins, we were intrigued by the identification of Cmt1 (CMytC), Cmt2 (CmytB), and protein PS1 (CMytA), all of which are mycoloyltransferase enzymes that have essential functions in mycomembrane biosynthesis and that were predicted in prior work to be mycomembrane-associated^37^ but have not previously been found to be *O*-mycoloylated. Beyond the porins and mycoloyltransferases, a number of other proteins with likely cell envelope-related locations and functions were identified (Supporting Table S6). The possible *O*-mycoloylation of mycoloyltransferases and other proteins of interest is discussed in more depth below in the additional context of PTM site mapping data.

To validate that O-AlkTMM labels proteins at *bona fide O*-mycoloylation sites, we performed additional targeted experiments on PorB, which is the most well-characterized *O*-mycoloylated protein that was identified in our O-AlkTMM-enabled LC-MS/MS screen. PorB was first reported to be *O*-mycoloylated by us in 2016^25^ and subsequently found to be dimycoloylated at Ser-7 and Ser-98 residues by the Renault group.^7^ We constructed Cg strains constitutively expressing His-tagged variants of either WT PorB or, alternatively, PorB with both Ser-*O*-mycoloylation sites mutated to Ala residues. These strains or the empty vector control were treated with O-AlkTMM and subjected to protein extraction as described above, followed by CuAAC reaction with azido-488, enrichment of His-tagged proteins on Ni^2+^-NTA resin, and SDS-PAGE analysis. Whereas O-AlkTMM robustly labeled His-tagged WT PorB, fluorescence was completely abolished in the Ser → Ala double mutant (Figure 2F). Thus, in the absence of known Ser-*O*-mycoloyl acceptor sites, O-AlkTMM did not label PorB. Taken together, our LC-MS/MS proteomic profiling data and follow-up validation of site-specific labeling of PorB demonstrate the ability of O-AlkTMM to reveal new insights into protein *O*-mycoloylation on the whole-proteome level.

### Development of Cleavable Linker and LC-MS/MS Strategies to Enable Site Mapping of Protein *O*-Mycoloylation

We next sought to create suitable strategies to comprehensively elucidate the Cg protein *O*-mycoloylome through proteome-wide protein identification and PTM site mapping, which necessitated the development of methods customized for the analysis of O-AlkTMM-labeled peptides. We attempted but failed to identify ester-linked modification sites from LC-MS/MS study 1 (Az-T-B) data set described above, possibly due to the large size of the Az-T-B modification, the HCD fragmentation method, or a combination of both. Therefore, we turned to affinity enrichment reagents with cleavable linkers, which have been developed to enable the selective release of biomolecules from beads while leaving a low-mass tag on the modified peptide to facilitate PTM site localization.^18,34^ Cleavable linkers also facilitate the elution of biotinylated proteins or peptides from streptavidin beads, which can be challenging due to the high affinity of the biotin–streptavidin interaction.^18,34^ A recent comparative analysis suggested that cleavable linkers outfitted with HCO_2_H-cleavable DADPS functional groups were advantageous for proteomics applications.^40^ Indeed, cleavable enrichment reagents containing DADPS between a clickable azido group and a biotin affinity tag (Az-DADPS-B)^33^ have been used broadly for PTM and protein interaction site identification.^41−44^ In addition to implementing a cleavable linker-based enrichment strategy, we also developed an LC-MS/MS approach that was designed to allow the analysis of the lipophilic *O*-acylated peptides generated through O-AlkTMM labeling. Specifically, we employed our recently reported LC method to separate hydrophobic peptides^45^ and an MS/MS method utilizing both HCD and AI-ETD fragmentation methods. AI-ETD aids in detecting labile peptide modifications,^46−49^ such as the ester-linked lipidic moiety generated through our chemical enrichment strategy. Based on the above considerations, we designed the strategies depicted in Figure 2Aii to enable robust proteome-wide identification and site mapping of *O*-mycoloylated proteins.

**Figure 3:**
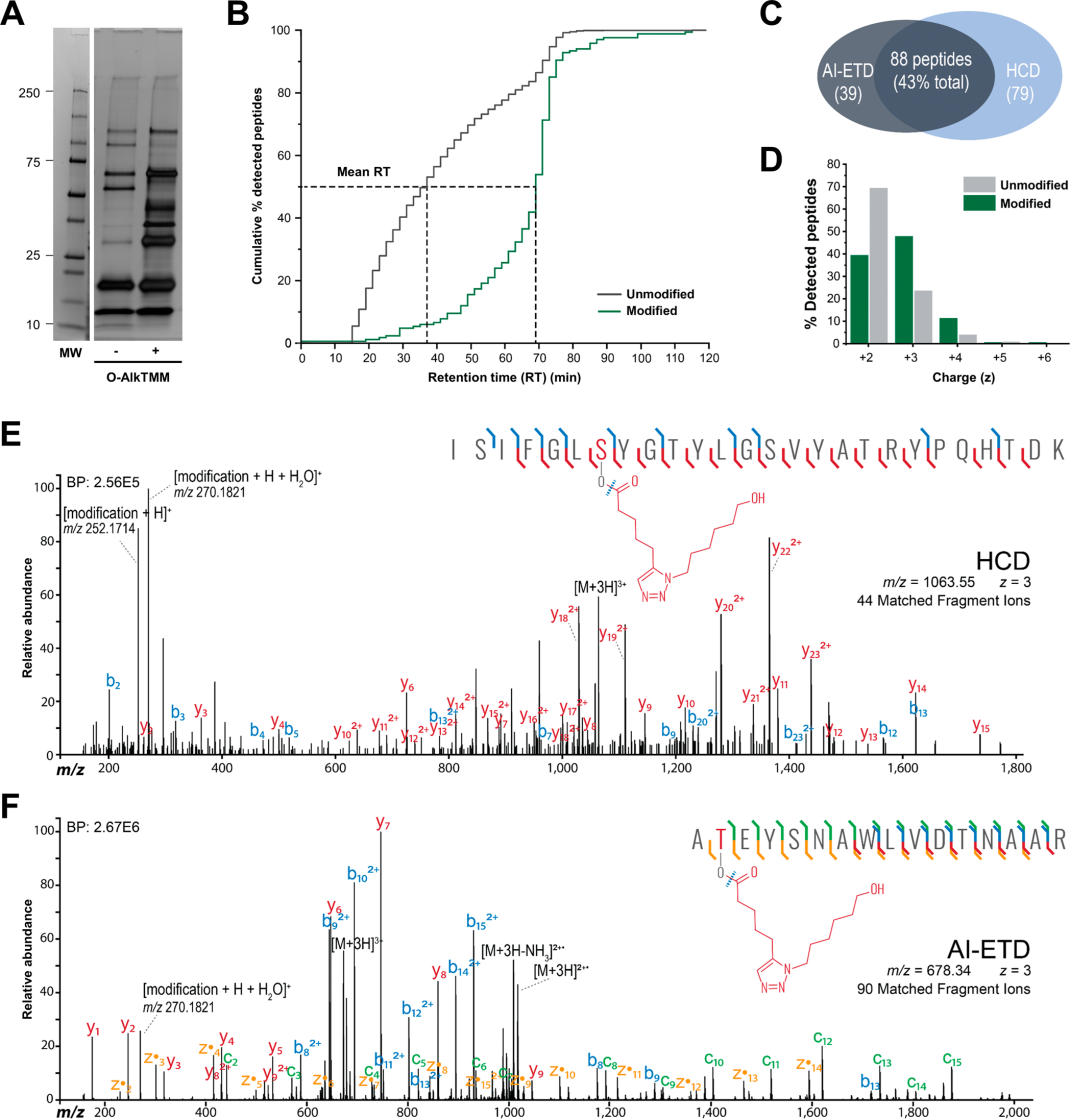
Identification of modified peptides using cleavable linker and LC-MS/MS strategies. (A) As depicted in Figure 2Aii, lysates from O-AlkTMM-treated Cg WT were subjected to CuAAC with Az-DADPS-B, and then the biotinylated proteins were captured on streptavidin beads. Beads were treated with 5% HCO_2_H to cleave the DADPS linker and release intact proteins, which were analyzed by SDS-PAGE with silver staining. (B–F) Data from LC-MS/MS study 2 (DADPS) and protein-level enrichment of O-AlkTMM-modified proteins. Proteins were enriched with O-AlkTMM and Az-DADPS-B as described in (A) and digested to generate modified peptides, which were then analyzed by a custom LC-MS/MS method. (B) Cumulative percent of the number of tryptic modified (gray) and unmodified (green) peptides detected at various retention times over 120 min. (C) Venn diagram of unique modified peptides detected by AI-ETD and HCD analyses. (D) Charge state distributions of unique modified (gray) and unmodified (green) peptides. (E, F) Examples of annotated (E) HCD and (F) AI-ETD spectra showing thorough sequence coverage and the presence of characteristic ions at *m*/*z* of 252.17 and 270.18, corresponding to the protonated modification with and without an adducted water molecule. Lists of ions for these spectra are given in Supporting Tables S12 and S13. Additional annotated spectra are shown in Supporting Figure S4.

First, we established conditions for the enrichment of O-AlkTMM-modified proteins using the DADPS-cleavable linker. Cg WT was treated with O-AlkTMM, and then alkyne-labeled proteins were extracted and reacted with Az-DADPS-B under CuAAC conditions. Click-biotinylated proteins were incubated with streptavidin-coated beads, which were subsequently washed and treated with 5% HCO_2_H to cleave the DADPS linker and release the proteins. Analysis of the bead output samples by SDS-PAGE with silver staining showed clear enrichment of proteins from O-AlkTMM-treated Cg cells compared to those of untreated control cells (Figure 3A). The protein profile of the output sample was virtually identical to that observed when using Az-T-B-based protein enrichment (Figure 2D), demonstrating consistency between these two enrichment approaches. This result confirmed that the DADPS cleavable linker enabled the biotinylation, capture, and selective release of O-AlkTMM-modified proteins obtained from Cg cells.

Next, we developed an LC-MS/MS method to separate and analyze the modified peptides obtained through DADPS-enabled, protein-level chemical enrichment. Briefly, O-AlkTMM-labeled proteins from Cg WT were reacted with Az-DADPS-B and enriched on streptavidin beads as described above and then subjected to on-bead digestion with trypsin. After unmodified peptides were washed away, the modified peptides that remained bound to the beads were released through the cleavage of the DADPS linker with 5% HCO_2_H and subjected to LC-MS/MS analysis. We recognized that the ester-connected modification on the peptide is quite hydrophobic and structurally reminiscent of a fatty acyl group—properties that would likely affect retention and elution in LC separations—and thus we developed an LC-MS/MS method to enable the efficient detection of these particular modified peptides. We employed our recently reported multiomic single-shot technology (MOST) for integrated proteome and lipidome analysis.^45^ Due to the optimized composition of mobile phases, MOST permits efficient separation of peptides and lipids in a single LC experiment and should be uniquely suitable for analyzing lipidated peptides. As expected, LC-MS/MS analysis employing MOST revealed that modified peptides exhibited considerably longer retention times (RTs) than unmodified peptides, with average RTs of 69.2 and 38.1 min, respectively (Figure 3B). While half of all modified peptides eluted after 69 min, only ∼15% of unmodified peptides eluted after 70 min. Furthermore, over 30% of the modified peptides eluted in the region of the chromatogram after 74 min, where virtually no unmodified peptides were observed.

To ensure efficient sequence coverage of enriched modified peptides, we deployed in parallel two distinct and complementary fragmentation strategies, HCD^50^ and AI-ETD.^51,52^ Owing to its speed and ease of implementation, HCD is a universally employed fragmentation technique. Meanwhile, AI-ETD has shown much promise for the fragmentation of modified peptides^46−49^ and nucleic acids.^53^ In the case of modified peptides obtained from O-AlkTMM-labeled proteins from Cg WT, both techniques successfully fragmented modified peptides and localized the modification sites, with 167 and 127 unique peptides detected by HCD and AI-ETD, respectively (Figure 3C). As illustrated by the observation that in common both techniques sequenced only 43% of all detected peptides, the two fragmentation approaches complemented each other by providing access to a unique subset of peptides and boosting the total number of detected modified peptides. We also observed that the modified peptides were, on average, more highly charged than the unmodified peptides (Figure 3D), likely because the modification retains a proton and hence is positively charged in the gas phase. In both HCD and AI-ETD spectra, we identified the formation of singly charged characteristic ions at *m*/*z* values of 252.17 and 270.18, corresponding to the protonated modification with and without an adducted water molecule. These ions could be utilized as diagnostic or trigger ions in future studies of this modification and peptides carrying it. Representative annotated spectra for peptides modified at Ser and Thr residues that were detected by HCD and AI-ETD, respectively, are shown in Figure 3E,F (lists of ions observed in these spectra are given in Supporting Tables S12 and S13; additional annotated spectra are shown in Supporting Figure S4). Overall, these results showed that the developed LC-MS/MS method was capable of efficiently separating and characterizing O-AlkTMM-modified peptides.

The combination of DADPS-based protein-level enrichment and our custom LC-MS/MS method identified a total of 275 modified peptides from 66 individual proteins (Supporting Table S7). After filtration of the results to include only modified peptides that were identified in both probe-treated replicates but absent from both untreated replicates, 235 modified peptides and 56 proteins were identified (Supporting Table S8). This LC-MS/MS study is herein referred to as “LC-MS/MS study 2 (DADPS)”. Promisingly, modified peptides were nearly exclusively detected in samples from O-AlkTMM-treated Cg WT and not in untreated bacteria, and all modifications occurred on Ser or Thr residues. Between study 2 employing DADPS enrichment and study 1 employing Az-T-B enrichment described above, there was good overlap, yet complementary information arising from the two approaches. Out of the 21 proteins identified via Az-T-B enrichment, 12 (57%) were also identified in the protein-level DADPS enrichment study. Among the proteins overlapping between these two studies were the mycoloyltransferases Cmt1 (CMytC), Cmt2 (CmytB), and protein PS1 (CMytA), potential mycomembrane- and peptidoglycan-remodeling enzymes Cgl2353 and Cgl2889, and several uncharacterized proteins. Unique protein identifications of interest in LC-MS/MS study 2 (DADPS) included an additional mycoloyltransferase (Cmt4/CMytF), a surface layer protein with high similarity to mycoloyltransferases (Cgl0922), and several other secreted proteins with known cell envelope-related functions, such as AftC, LcpA, and bacterial lipocalin. These and other identified proteins’ biological functions, as well as the characteristics and potential significance of their *O*-mycoloylation status, are further discussed below. Although the DADPS enrichment study provided additional information about protein identity and modification sites, we also noted the detection of a number of modified peptides arising from proteins that are unlikely to be *O*-mycoloylated (e.g., ribosomal proteins, acyl CoA carboxylase, 2-methylcitrate synthase) (Supporting Table S8), which suggested the possibility of some Cmt1-independent labeling of proteins. In addition, we analyzed limited replicates (*n* = 2) of probe-treated and untreated Cg WT in study 2 since this study was done primarily for the purpose of LC-MS/MS method development. Therefore, we sought to increase the certainty of our identification and site mapping results.

### High-Confidence, Proteome-Wide Identification and Site Mapping of *O*-Mycoloylated Proteins

To provide high-confidence protein identification and site mapping information and to account for potential nonspecific attachment of O-AlkTMM to proteins, we performed a third LC-MS/MS study with additional controls and more stringent criteria for inclusion in our peptide/protein list. To confidently differentiate between authentic Cmt1-dependent modifications and possible non-Cmt1 modifications arising from O-AlkTMM treatment, we performed a parallel analysis in both Cg WT and Δ*cmt1* strains. We again used Az-DADPS-B to enrich O-AlkTMM-labeled proteins, as shown in Figure 2Aii, although in this third study, we used peptide-level enrichment. Peptide-level enrichment, in which alkyne-tagged proteins are first proteolytically digested and then subjected to CuAAC reaction and bead capture, has previously been reported to increase the efficiency of the enrichment of modified peptides as compared to protein-level enrichment.^54,55^ Thus, Cg WT or Δ*cmt1* cells were treated with O-AlkTMM or left untreated, and then proteins were collected and reacted with Az-DADPS-B under CuAAC conditions and digested using trypsin. The resulting biotinylated peptides were captured on streptavidin-coated beads, which were then washed extensively to remove unmodified peptides. Treatment of the beads with 5% HCO_2_H cleaved the DADPS linker, selectively releasing peptides bearing a low-mass modification at the PTM site. Modified peptides obtained from O-AlkTMM-treated or untreated Cg WT or Δ*cmt1* strains were analyzed using the custom LC-MS/MS method described above.

In total, LC-MS/MS analysis identified 179 modified peptides from 68 individual proteins in this study, which is referred to herein as “LC-MS/MS study 3 (DADPS)” (Supporting Table S9). To filter the list of modified peptides from study 3 down to high-confidence hits, we included in the curated list only modified peptides that were identified in at least 3 of 4 O-AlkTMM-treated Cg WT replicates, and we excluded any modified peptides that were either (i) identified in at least 2 of 3 untreated control replicates or (ii) identified in at least 2 of 3 O-AlkTMM-treated Δ*cmt1* mutant control replicates. This allowed for the removal of identifications that may have resulted from Cmt1-independent, nonspecific labeling of proteins by O-AlkTMM. Following this filtration process, we included 59 modified peptides correlating to 27 individual proteins in our curated list for study 3 (Tables 1 and S10). Approximately two-thirds of the modified peptides were exclusively identified in O-AlkTMM-treated Cg WT samples. For the remaining one-third, while there were very few modified peptides identified in untreated Cg WT or Δ*cmt1* samples, a modest number of modified peptides were detected in O-AlkTMM-treated Cg Δ*cmt1*. This result suggests that a small amount of Cmt1-independent labeling occurred, which could be due to nonenzymatic probe incorporation and/or incorporation by other mycoloyltransferase isoforms or other acyltransferases. This result is consistent with the faint residual fluorescence observed by SDS-PAGE analysis of O-AlkTMM-treated Cg Δ*cmt1* lysates (Figure 2A). The large majority of proteins that were excluded from our list based on modified peptide identification in the Δ*cmt1* mutant were probable cytoplasmic proteins that are very unlikely to be *O*-mycoloylated (Supporting Tables S9 and S10). It is possible that filtration of results using the Δ*cmt1* mutant could obscure some authentic *O*-mycoloylated proteins that might be generated by other mycoloyltransferase isoforms, although this is not a significant concern since only Cmt1 has been demonstrated to have protein *O*-mycoloyltransferase activity and our data show that the overwhelming majority of protein labeling in Cg is Cmt1-dependent (Figure 2A). Overall, our results indicate that the Δ*cmt1* control and more stringent inclusion criteria significantly improved the reliability of the peptide and protein identifications in study 3. It is also notable that the number of modified peptides identified via peptide-level DADPS enrichment used in study 3 was comparable to that via protein-level DADPS enrichment in study 2.

**Table 1:** Protein and Modification Site Identifications, Curated from LC-MS/MS Study 3 (DADPS)^a^

function	gene name	protein description	modification site(s)
cell wall synthesis and remodeling	cmt2	Cmt2 (CMytB)	T_98_, S_101_, T_225_, S_236_, S_204_, S_180ca_^b^
	csp1	protein PS1 (CMytA)	S_103_, S_132_, S_517_, S_226ca_^b^
	cmt1	Cmt1 (CMytC)	S_281_, S_284_, S_296_
	aftC	α-(1 → 3)-arabinofuranosyl transferase	S_275_, S_278_, S_279_
	Cgl2353	PE–PPE domain-containing protein	S_105_, S_150_
	Cgl1538	cell wall-associated hydrolases (invasion-associated proteins)	S_502_
	Cgl2008	cell division protein FtsI/penicillin-binding protein 2	S_534_
hydrolases	Cgl1391	SGNH_hydro domain-containing protein	S_45_, S_46_, S_227_, S_229_
	Cgl1093	putative peptidase Cgl1093	T_40_
	Cgl1714	peptidase S1 domain-containing protein	S_335_
others	Cgl0628	bacterial lipocalin	S_39_, S_40_, S_43_, S_46_, S_138_, S_165_
	ctaC	cytochrome c oxidase subunit 2	S_201_, S_203_
	qcrC	cytochrome bc1 complex cytochrome c subunit	S_191_, S_192_
	Cgl2461	ABC-type transporter, periplasmic component	S_71_
	Cgl1217	lactoylglutathione lyase and related lyases	T_126_
uncharacterized proteins	Cgl1342	uncharacterized protein	S_137_, S_138_
	Cgl0524	uncharacterized protein	S_67,_ T_66_
	Cgl1696	uncharacterized protein	S_127_, S_133_
	Cgl0835	uncharacterized protein	S_279_, S_280_
	Cgl0651	uncharacterized protein	S_226_
	Cgl1840	uncharacterized protein	S_397_
	Cgl2546	uncharacterized protein	S_119_
	Cgl2912	uncharacterized protein	S_271_
	Cgl0559	uncharacterized BCR	T_87_
	Cgl0125	hypothetical membrane protein	S_439_
	Cgl3015	hypothetical membrane protein	S_54_
	Cgl2760	hypothetical membrane protein	S_228_

a Modification sites were included if detected in at least 3 of 4 O-AlkTMM-treated Cg WT replicates and absent in at least 2 of 3 replicates of the negative controls, including O-AlkTMM-treated Cg Δ*cmt1* and untreated WT or Δ*cmt1*. See Supporting Tables S9 and S10 for corresponding raw and curated LC-MS/MS data.

b Modification sites denoted as “ca” are known catalytic Ser residues. See the Discussion section.

**Figure 4:**
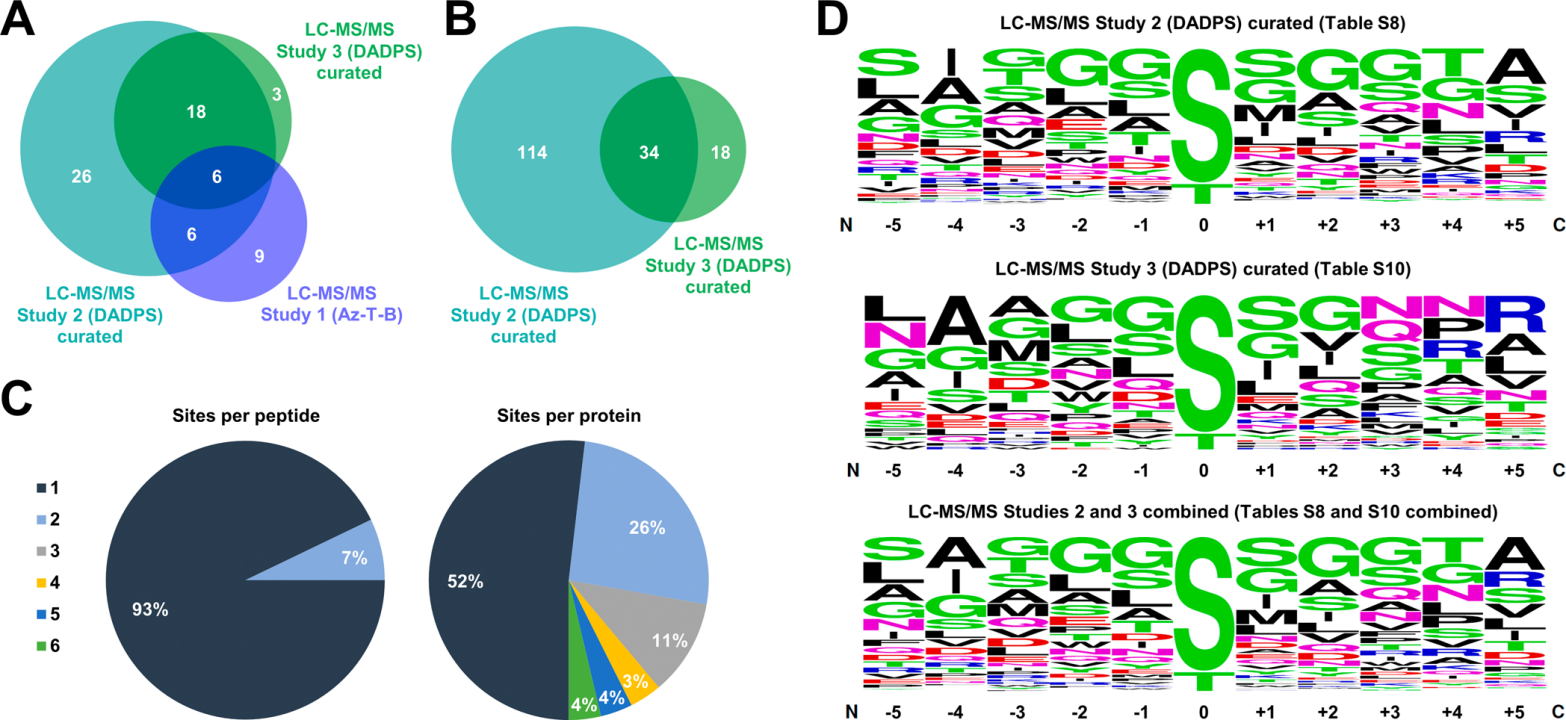
Characteristics of modified proteins determined by LC-MS/MS identification and site mapping studies. (A) Venn diagram comparison of proteins identified in LC-MS/MS studies 1–3. (B) Venn diagram comparison of the number of modification sites identified in LC-MS/MS studies 2 and 3. (C) Distribution of the number of modification sites observed per peptide and per protein in LC-MS/MS study 3. (D) Results of motif enrichment analysis conducted using WebLogo using data from LC-MS/MS study 2 (top), study 3 (middle), and the combined data from studies 2 and 3 (bottom). All data for LC-MS/MS studies 2 and 3 in panels (A–D) are from the curated lists (Tables 1, S8 and S10).

Out of the 27 proteins identified with high confidence via peptide-level enrichment in LC-MS/MS study 3 (DADPS) (Table 1), 24 (89%) were also identified via protein-level enrichment in LC-MS/MS study 2 (DADPS), indicating excellent overlap between the two DADPS enrichment studies with respect to protein identification (Figure 4A and Supporting Table S11). Accordingly, the proteins identified in study 3 (Table 1) were similar to those identified in study 2 and included multiple mycoloyltransferase isoforms, cell envelope biosynthesis and remodeling enzymes, and other proteins of interest, whose functions and *O*-mycoloylation status are further discussed below. As anticipated, study 3 had a substantially lower number of obviously off-target identifications than study 2 due to the additional controls and cutoff criteria (Supporting Tables S10 and S11). In addition, there was a good overlap between the two DADPS studies with respect to the putative *O*-mycoloylation sites identified, as 34 of the 52 (65%) modification sites identified in study 3 were also identified in study 2 (Figure 4B). Of note, study 3 exhibited a lower overlap with the 21 proteins identified in LC-MS/MS study 1 (Az-T-B), among which only 6 (26%) were also identified in study 3; nonetheless, these 6 proteins were identified in all 3 LC-MS/MS studies, and their known functions all relate to mycomembrane synthesis and remodeling (Supporting Table S11). In addition, among the 19 proteins previously predicted to be mycomembrane-associated in Cg by Marchand et al., our three LC-MS/MS studies identified 16 proteins (84%) as *O*-mycoloylated, showing excellent overlap and strongly supporting the hypothesis that *O*-mycoloylation targets proteins to the mycomembrane (Supporting Figure S3B). In total, 30 proteins were identified as putatively *O*-mycoloylated in at least 2 out of the 3 LC-MS/MS studies conducted, including 24 identified with high confidence in study 3, which provides a reliable list to analyze and base future characterization studies on.

We analyzed the high-confidence curated data from LC-MS/MS study 3 (DADPS) (Supporting Table S10) to gain insight into the nature of the *O*-mycoloyl modification. We observed that ∼93% of the modified peptides contained a single *O*-mycoloylation site, whereas the remaining ∼7% of peptides carried 2 modifications (Figure 4C). On the protein level, approximately half of the detected proteins (52%) harbored only one modification site. Meanwhile, nearly half of the proteins (48%) contained 2 or more modifications, with ∼20% of proteins being heavily decorated with 3 or more *O*-mycoloylation sites. This observation is consistent with prior work, which demonstrated that even low-molecular-weight *O*-mycoloylated proteins from a Cg chloroform–methanol extract may contain multiple *O*-mycoloyl groups, including some with as many as 5 modifications.^7,25^

We also performed motif analysis^56^ on the site mapping data from LC-MS/MS studies 2 and 3, aiming to uncover a potential recognition sequence surrounding the modified amino acids (Figure 4D). Similar to the protein identification results, there was excellent agreement between studies 2 and 3 with respect to the sequence motif analysis. The modification was exclusively identified on Ser and Thr residues and not any other amino acids, including other nucleophilic residues that could potentially react with O-AlkTMM (e.g., Tyr, Lys, Cys, and Arg). Most modifications were observed on Ser residues, with Thr being modified at ∼1/9th of the rate. The sequence surrounding the Ser/Thr modification site exhibited slight enrichment for relatively small and in some cases hydrophobic amino acids, such as glycine, alanine, and serine. These findings provide evidence that *O*-mycoloylation may occur in Ser-rich regions and/or next to smaller hydrophobic amino acids, potentially to minimize steric hindrance and accommodate the large and lipophilic *O*-mycoloyl group.

**Figure 5:**
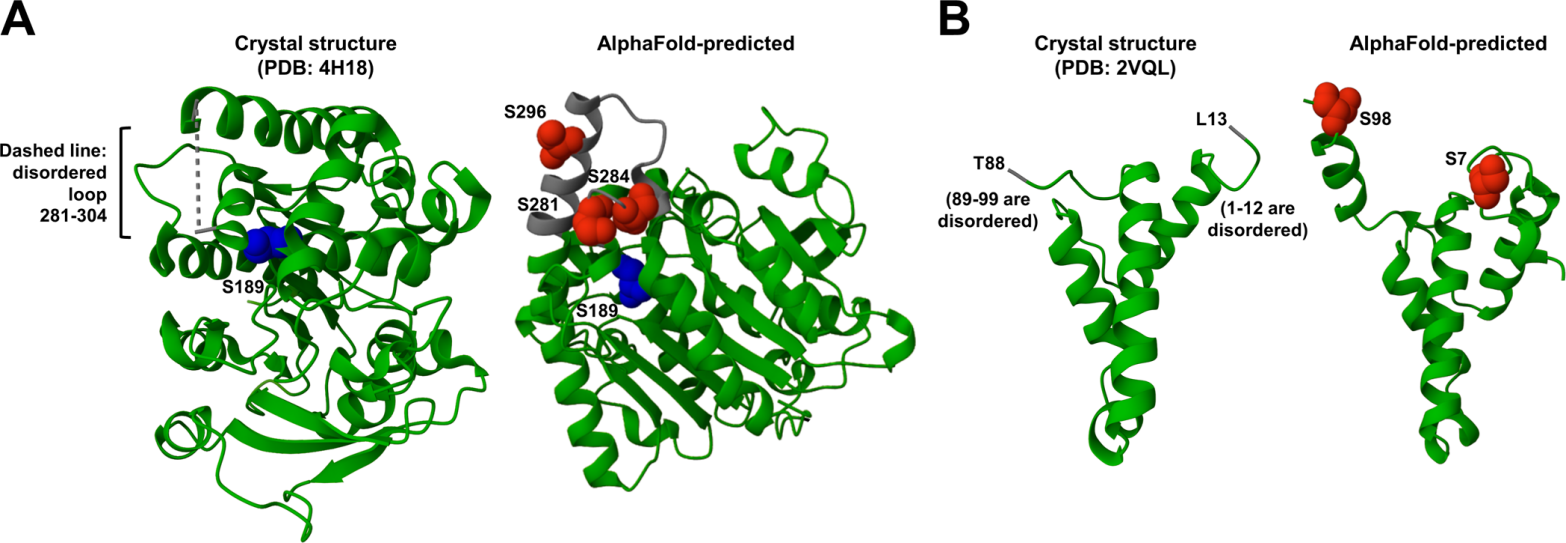
Modification sites are spatially clustered on multiply modified proteins. (A, left) Crystal structure of Cmt1 (PDB ID: 4H18). All putative *O*-mycoloylation sites on Cmt1 were identified within the highlighted disordered loop consisting of residues 281–304. Catalytic site, blue. (A, right) AlphaFold structure of Cmt1. Disordered loop in crystal structure, gray; modification sites, red; catalytic site, blue. (B, left) Crystal structure of PorB (PDB ID: 2VQL). *O*-Mycoloylation sites on PorB were identified within the disordered regions at the termini. (B, right) AlphaFold structure of PorB. Modification sites, red. Note: the signal sequences for both AlphaFold-predicted structures were removed. See Supporting Figure S5 for the AlphaFold-predicted structures of other candidate *O*-mycoloylated proteins.

The finding that many *O*-mycoloylated proteins have multiple putative lipidation sites, which is consistent with our prior work on Cg porins,^25^ raises questions about the spatial distribution of the sites on multiply modified proteins. Among the proteins we identified as *O*-mycoloylated with high confidence (Table 1), Cmt1 has a reported 3D structure, which is in its nonacylated form (PDB ID: 4H18(30)). Interestingly, the 3 putative modification sites we identified in Cmt1 (Ser-281, Ser-284, and Ser-296) are all located on a short, surface-exposed, and disordered loop spanning residues 281–304 (Figure 5A, left). The close spatial proximity of the modifications in Cmt1 was evident in an AlphaFold-predicted^57^ structure (Figure 5A, right). Consistently, the crystal structure of PorB in nonacylated form (PDB ID: 2VQL) was also disordered near the N- and C-termini, where the two *O*-mycoloylation sites occur at Ser-7 and Ser-98 (Figure 5B, left). In the AlphaFold-predicted^57^ structure of PorB, the two modification sites are close to one another in space (Figure 5B, right). In the cases of both Cmt1 and PorB, the spatially clustered nature of the modifications, along with the lack of residue conservation surrounding the modification site (Figure 4D), further suggests that perhaps sterically accessible Ser-rich regions of protein substrates may be a more important determinant for *O*-mycoloylation than a consensus amino acid sequence. In these two examples, it is also apparent that the lipid modifications likely originate from one face of the protein, which would allow the protein to be oriented such that multiple *O*-mycoloyl groups could insert into the mycomembrane simultaneously. Given that the 3D crystal structures of the nonacylated forms of Cmt1 and PorB exhibit disorder in their lipidated regions, it is intriguing to consider whether *O*-mycoloylation would lead to stabilization of these regions. We also mapped the identified putative *O*-mycoloylation sites onto AlphaFold-predicted^57^ 3D structures of all other candidate proteins and found that, like Cmt1 and PorB, many proteins exhibited putative lipidation sites clustered together (Supporting Figure S5). As additional experimentally determined 3D structures of mycoloyltransferases, porins, and other identified proteins become available, it will be of high interest to analyze the location and context of their putative *O*-mycoloylation sites revealed in our study.

## Discussion

### Insights into Bacterial Protein *O*-Mycoloylation Revealed by Chemical Proteomics

Protein *O*-mycoloylation is a unique PTM that was first discovered to occur on small hydrophobic porins in Cg, which facilitate the passage of small molecules across the notoriously impenetrable mycomembrane.^5^ Subsequent studies suggested that Cg porins can harbor multiple Ser-*O*-mycoloyl modifications and that the PTM may target these proteins to the mycomembrane, anchoring them to this cell envelope layer to perform their channel functions.^7,25^ Yet, several important knowledge gaps regarding the scope and functions of this unique PTM remained. Prior studies on the initial discovery and characterization of *O*-mycoloylated proteins were limited, focusing on a few porins that are enriched in a chloroform–methanol extract of Cg. Such a targeted approach necessarily precludes the identification of additional proteins carrying this PTM that may exist in the Cg proteome. However, as discussed in the Introduction section, proteomic profiling of lipid PTMs—especially the large and highly hydrophobic *O*-mycoloyl PTM—is challenging due to the inherent difficulty of detecting lipidated peptides.

To address these challenges, we enlisted chemical proteomics, which harnesses the selective chemical labeling of proteins of interest to enable downstream analysis on the proteome scale.^58^ By combining O-AlkTMM metabolic labeling with click chemistry, we developed strategies to specifically visualize, enrich, identify, and characterize the modification sites of *O*-mycoloylated proteins (Figure 2A). We first found that *O*-mycoloylation occurs on numerous proteins beyond the few low-molecular-weight porins previously confirmed to be modified. Then, we carried out multiple LC-MS/MS studies to identify these proteins and map their modification sites. Our first study, LC-MS/MS study 1 (Az-T-B), used conventional biotin–streptavidin enrichment and LC-MS/MS methods to identify O-AlkTMM-labeled proteins (Figure 2Ai), which led to the first protein list comprising the putative Cg protein *O*-mycoloylome (Figure 2E and Supporting Tables S4–S6). We next aimed to improve the coverage and confidence of protein identifications as well as to obtain critical PTM site mapping information. This was achieved using the Az-DADPS-B cleavable biotinylation reagent and an LC-MS/MS method customized for the detection of lipidated peptides (Figure 2Aii). Importantly, these methods enabled the efficient enrichment, separation, and identification of peptides bearing a fatty acyl-like modification with a modest size and defined mass at the *O*-mycoloylation site, thus addressing the challenges associated with detecting native lipidated peptides in complex samples. After establishing these methods through LC-MS/MS study 2 (DADPS) (Figure 3 and Supporting Tables S7 and S8), we applied the methods along with additional controls and stringent inclusion criteria in LC-MS/MS study 3 (DADPS), which generated high-confidence protein identification and site mapping data (Table 1 and Supporting Tables S9 and S10) that is the major basis of the following discussion.

One significant finding provided by our proteomic data sets is the identification of numerous candidate *O*-mycoloylated proteins. As shown in Table 1, many of the proteins identified as putatively *O*-mycoloylated are secreted proteins with either known or predicted functions related to the cell envelope and, more specifically, the mycomembrane. Among the most intriguing findings is that multiple Cg mycoloyltransferases were identified as candidate *O*-mycoloylated proteins. Mycoloyltransferases, which are abundant, conserved, and collectively essential for viability in *Corynebacterineae* species, catalyze the transfer of mycoloyl groups from the donor TMM onto acceptor molecules to generate the major mycomembrane glycolipids, AGM and TDM, as well as *O*-mycoloylated proteins (Figure 1A).^27,28^ Presumably, mycoloyltransferases must localize within or near the mycomembrane to perform these critical biosynthetic functions. Consistent with this idea, mycoloyltransferases in various *Corynebacterineae* species have been found to be associated with the cell envelope.^28^ In addition, mycoloyltransferases are found at high levels in the culture medium in soluble, nonacylated forms.^28^ In the context of pathogens, mycoloyltransferases are known to be immunogenic and have been exploited in tuberculosis diagnostic and vaccine development efforts.^59^

Previously, it has been postulated that mycoloyltransferases may be post-translationally modified,^7,28,60−62^ which could possibly explain their ability to associate with the cell envelope. However, to date, these enzymes have not been demonstrated to be modified, and their mechanism of cell envelope association remains unclear. Our LC-MS/MS data provide the first experimental evidence that Cg mycoloyltransferases may be modified, specifically by *O*-mycoloyl groups. Three Cg mycoloyltransferase isoforms, Cmt1 (CMytC), Cmt2 (CMytB), and protein PS1 (CMytA), were identified as *O*-mycoloylated in all three of our LC-MS/MS studies. Furthermore, in LC-MS/MS study 2, we identified an additional known mycoloyltransferase, Cmt4 (CMytF), as well as Cgl0922, which is annotated as a surface layer protein but has a high sequence similarity to mycoloyltransferases in Cg and related organisms. Together, these proteins account for almost all of the mycoloyltransferases encoded by the Cg genome. Our PTM site mapping data suggest that Cg mycoloyltransferases are highly *O*-mycoloylated, with each having ≥3 modification sites. Taken together, these data suggest a scenario in which Cmt1-dependent *O*-mycoloylation of mycoloyltransferases may enable targeting of these proteins to, and association with, the Cg mycomembrane to perform biosynthetic functions. An intriguing implication of the observed modifications of Cmt1 is that this enzyme may modify itself through autocatalytic *O*-mycoloylation, which, along with possible implications for mycomembrane protein targeting, necessitate further investigation. Given that mycoloyltransferases are also present in the extracellular culture medium in nonacylated forms, the *O*-mycoloyl modification could be playing a role in regulating the location and activity of mycoloyltransferases, and potentially other modified proteins—Cg porins are also found in nonacylated, extracellular forms in addition to their acylated, mycomembrane-associated forms.^7^ If this is the case, it remains unclear whether some proportion of protein is exported from the cell without undergoing post-translational *O*-mycoloylation or protein *O*-mycoloylation is a dynamic PTM that can be reversed through the action of an as-yet unidentified esterase(s), or a combination of both. Indeed, the precise sequence and location of protein *O*-mycoloylation (and possibly de*-O*-mycoloylation) events are not yet known and require further research.

We also observed that the catalytic Ser residues of the mycoloyltransferases Cmt2 and protein PS1 were detected as modified, which is consistent with the O-AlkTMM incorporation mechanism being dependent on mycoloyltransferase activity, as depicted in Figure 1B. First, this finding provides direct evidence of the specificity and proposed mechanism of O-AlkTMM incorporation and, more generally, of native TMM and other reported TMM-based probes^20^ that rely on mycoloyltransferase activity for incorporation into mycomembrane glycolipids and proteins. Second, the result suggests that catalytic sites of some mycoloyltransferases can be stably covalently modified by O-AlkTMM in the form of a trapped acyl-enzyme intermediate, an observation that may be of interest for the future development of tools to inhibit or investigate the mechanism of mycoloyltransferases. It is unlikely that these active site modifications represent authentic *O*-mycoloylation sites. Indeed, many of the peptides identified with a modified catalytic Ser residue were detected in the Δ*cmt*1 mutant, demonstrating that these modifications occurred independently of Cmt1. It is possible that residual fluorescence observed in lysates collected from O-AlkTMM-treated Cg Δ*cmt*1 mutant (Figure 2B) could be due in part to active site labeling of mycoloyltransferases. Regardless, these results underscore the value of filtering out Cmt1-independent O-AlkTMM-labeled peptides by leveraging the Cg Δ*cmt*1 mutant, as we did in LC-MS/MS study 3 to attain the high-confidence hit list presented in Table 1. Notably, the active site Ser residues of the mycoloyltransferases Cmt1 and Cmt4 were not detected as modified by O-AlkTMM.

In addition to mycoloyltransferases, a number of other cell envelope-related proteins were identified as putatively *O*-mycoloylated, several with functionally important orthologs in pathogenic organisms (Supporting Table S10). For example, although Cgl2353 has not previously been directly studied in Cg to the best of our knowledge, it is annotated as a PE–PPE domain-containing protein, a class of proteins that in *M. tuberculosis* have been shown to have roles in virulence and immunomodulation, and in some cases to be mycomembrane-associated.^63−65^ The closest ortholog of Cgl2353 in *M. tuberculosis* is Rv3451, a characterized TDM hydrolase that remodels the mycomembrane to balance nutrient acquisition and stress tolerance as the environment changes.^66^ In the model organism *Mycobacterium smegmatis*, TDM hydrolase (MSMEG_1529) activity is essential for biofilm formation and the protein was identified as mycomembrane-resident.^67−69^ Another cell envelope-related hydrolase identified as a likely *O*-mycoloylated protein is Cgl1538, whose orthologs in *M. tuberculosis*, RipA and RipB, hydrolyze peptidoglycan during cell division, are involved in macrophage invasion, and are required for persistence in mice.^70^ Also identified was AftC, an α(1 → 3)-arabinofuranosyl transferase implicated in the biosynthesis of the cell wall-associated glycolipids arabinogalactan and lipoarabinomannan.^71^ Other predicted secreted hydrolases identified as *O*-mycoloylated include Cgl1391, Cgl1093, and Cgl1714, all of which will be of interest to study for possible cell envelope synthesis or remodeling functions. Bacterial lipocalin, which localizes to and is involved in the maintenance of bacterial outer membranes,^72^ was also identified and possesses numerous putative *O*-mycoloylation sites. Another dozen uncharacterized proteins, including hypothetical membrane proteins, were also identified as putatively *O*-mycoloylated and represent candidates for future investigation. Overall, many of the proteins identified have established or predicted cell envelope functions that could conceivably be facilitated and regulated by *O*-mycoloylation-dependent association with the mycomembrane. Indeed, among the 16 candidate *O*-mycoloylated proteins with functional annotations, half were previously suggested as possible mycomembrane-associated proteins based on cellular fractionation experiments that enriched for a mycomembrane-containing cell wall fraction (Table S10).^37^ Moreover, when considering all of the proteins identified as possibly *O*-mycoloylated through LC-MS/MS studies 1–3, these remarkably include 16 of the 19 proteins (84%) previously predicted to reside in the mycomembrane (Supporting Figure S3B).

There are potential limitations to our approach that are important to take into consideration with respect to protein identifications. First, the identifications are based on the modification of protein substrates by an unnatural chemical probe, O-AlkTMM, rather than the native metabolite, TMM. Our study demonstrates a high level of Cmt1-dependent O-AlkTMM incorporation into proteins and validates site-specific labeling of PorB, which together provide confidence that O-AlkTMM is on target. However, it is possible that (i) simplifications to the probe structure relative to the native substrate (e.g., shortened, unbranched chain) and/or (ii) the probe administration conditions (exogenously added; probe concentration may not reflect native substrate concentration) could affect the protein profile. For example, we did observe minor Cmt1-independent incorporation of O-AlkTMM into proteins that could arise either from other mycoloyltransferases/acyltransferases or from nonenzymatic labeling. It will be of interest in the future to perform targeted follow-up studies to validate the native lipidation of candidate *O*-mycoloylated proteins newly identified in this study, such as mycoloyltransferases. Second, we did not identify porins in our high-confidence list obtained from LC-MS/MS study 3, despite their validated *O*-mycoloylation status and the ability of O-AlkTMM to robustly metabolically label them (ref (25) and Figure 2F). As mentioned above, this is likely due to the inherent limitations of using bottom-up proteomics to detect small, hydrophobic proteins containing few to no tryptic digestion sites.^38^ Thus, we acknowledge that top-down LC-MS/MS analysis of intact proteins may be preferable for the targeted study of purified low-molecular-weight *O*-mycoloylated porins,^7^ whereas our bottom-up LC-MS/MS strategies are more suitable for unbiased and proteome-wide discovery, identification, and site mapping of larger *O*-mycoloylated proteins. On the other hand, we detected PorB and PorH as *O*-mycoloylated proteins in LC-MS/MS study 1 using Az-T-B enrichment (Figure 2E), so it is possible that optimization of protein extraction and enrichment methods can maximize coverage. The combination of complementary, optimized LC-MS/MS approaches should enable holistic analysis of probe-modified or native *O*-mycoloylated proteins of diverse size. Toward these goals, the chemical, genetic, and LC-MS/MS tools and methods reported here, which are tailored to facilitate the detection and characterization of *O*-acylated lipophilic peptides, should prove beneficial.

In addition to significantly extending the scope of the known protein *O*-mycoloylome, the reported proteomic data sets have revealed characteristics of the *O*-mycoloylation modification through global PTM site mapping. Previously reported studies focused on low-molecular-weight porins in Cg identified 6 PTM sites across 4 proteins,^7^ which yielded important but limited information about the nature of the modification. To these data, our study adds >50 putative PTM sites occurring across ∼30 individual proteins (Table 1), providing rich additional information about the modification. The modification occurs at Ser or—less frequently—Thr residues, generally among neighboring Ser and other relatively small and hydrophobic residues (e.g., Gly, Ala), although a strict consensus motif is absent (Figure 4D). As all previously characterized *O*-mycoloylation sites on Cg porins occur on Ser, this is the first evidence suggesting that Thr-*O*-mycoloylation may also occur. It is possible that the relatively flexible, accessible, and nonpolar peptide motif is necessary to permit Cmt1-catalyzed modification by the large and hydrophobic mycoloyl group. The lack of a strict consensus sequence also raises the possibility that an adapter protein(s) and/or colocalization with Cmt1 could be involved in the selection of polypeptides as substrates for *O*-mycoloylation. Notably, the lack of a consensus sequence may impede efforts to develop straightforward bioinformatic tools to identify *O*-mycoloylated proteins, further underscoring the importance of chemical labeling strategies. Approximately half of the proteins identified were found to contain more than one *O*-mycoloylation site, and several proteins contained ≥3 modifications (Figure 4C and Table 1). Our analysis of the location of modifications on experimentally determined and AlphaFold-predicted 3D structures suggests that multiply modified proteins may have clustered *O*-mycoloylation sites (Figures 5 and S5), which could have implications for the orientation and strength of the protein association with the mycomembrane.

There has been a long-standing interest in the identification and characterization of mycomembrane proteins in the *Corynebacterineae*, as these proteins are predicted to have critical functions in transport, cell envelope construction and maintenance, and host interactions.^14^ However, mycomembrane proteomes are severely undercharacterized compared to Gram-negative outer membrane proteomes, owing to the extraordinary complexity of the *Corynebacterineae* cell envelope and the lack of tools suited to the task.^14,15^ Given that protein *O*-mycoloylation appears to be a mechanism for targeting proteins to the Cg mycomembrane—a hypothesis further supported by the data presented herein—the reported strategy for specific labeling and analysis of *O*-mycoloylated proteins represents a valuable tool to gain insight into the mycomembrane proteome in Cg and related organisms. Indeed, the present work expands the list of putative mycomembrane-associated proteins in Cg to include those identified in Table 1. These results are consistent with data from recent studies using complementary cellular fractionation^37,73^ and photo-cross-linking approaches,^69^ both of which similarly identified porins, mycoloyltransferases, and secreted hydrolases as likely mycomembrane proteins.

It remains to be determined how widely the *O*-mycoloylation PTM occurs in bacterial species outside of Cg. Given that a Cg mutant lacking the protein *O*-mycoloylation machinery has a growth defect and is sensitized to antibiotics,^9^ it will be of particular interest to search for the PTM in pathogens. The protein *O*-mycoloyltransferase Cmt1 in Cg has closely related orthologs in the diphtheria-causing pathogen *C. diphtheriae* and several other species in the *Corynebacterineae* suborder, including within the genera *Corynebacterium*, *Nocardia*, and *Rhodococcus*.^30^ To date, protein *O*-mycoloylation has not been demonstrated to occur in mycomembrane-containing mycobacterial species of interest, such as the pathogen *M. tuberculosis* or the model organism *M. smegmatis*. Although mycobacteria lack strict orthologs of Cg Cmt1, they possess multiple structurally similar mycoloyltransferases with relatively high substrate tolerance that should be explored for protein *O*-mycoloylation activity. The tools reported herein will facilitate efforts to discover and characterize *O*-mycoloylated proteins in additional organisms and, along with other emerging strategies for studying cell envelope proteins in the *Corynebacterineae*,^16,74^ are expected to contribute to an improved understanding of the mycomembrane proteome. Our tools may also be useful for comparing and contrasting the scope and functional roles of *O*-mycoloylation and *N*-acyl-*S*-diacyl-glycerylation, the latter of which generates lipoproteins that associate with the cell envelope in corynebacteria and mycobacteria.^75,76^

As protein *O*-mycoloylation represents the only known example of protein *O*-acylation in bacteria, it is useful to consider our data on this PTM in a broader context. Protein *O*-acylation is known to exist in eukaryotic systems, including Ser-*O*-palmitoleoylation of Wnt proteins^4,77^ and Ser-*O*-octanoylation of ghrelin.^3,78,79^ The major function of these and other types of protein lipidation (e.g., *S*-palmitoylation, *S*-prenylation, *N*-myristoylation, *N*-acyl-*S*-diacyl-glycerylation) is to modulate protein interactions, predominantly for the purpose of directing and anchoring proteins to membranes.^2,76^ Similarly, the major function of protein *O*-mycoloylation appears to be targeting proteins specifically to the mycomembrane, as discussed above. The scope of eukaryotic protein *O*-acylation seems to be relatively limited, as PORCN-catalyzed *O*-palmitoleoylation is only known to occur on Wnt proteins^77^ and GOAT-catalyzed *O*-octanoylation only occurs on a single polypeptide substrate, ghrelin.^78,79^ By contrast, our data suggest that Cmt1-catalyzed *O*-mycoloylation in Cg is widespread, occurring on dozens of proteins of diverse size and biological function (Table 1). There may be some similarity between eukaryotic and bacterial *O*-acylation with respect to the modification site, as the Wnt-3a *O*-palmitoleoylation motif (GLSGS)^80^ and the ghrelin *O*-octanoylation motif (GSSFL)^78,79,81^ each exhibit multiple Ser residues and at least one Gly residue, which reflects the Ser- and Gly-rich *O*-mycoloylation motif revealed by our site mapping data (Figure 4D). However, unlike eukaryotic *O*-palmitoleoylation and *O*-octanoylation, which modify single Ser residues of target proteins, bacterial *O*-mycoloylation machinery frequently modifies the same protein at multiple sites, which appear to often be condensed in the same region (Table 1; and Figures 4C, 5 and S5). In this regard, protein *O*-mycoloylation more closely resembles *S*-palmitoylation, which can also occur on multiple sites of the same protein.^1^ Although the reason for multiple *O*-mycoloylations of single proteins remains unknown, one possibility is that more hydrophilic proteins require additional *O*-acylations to strengthen association with the mycomembrane. Finally, there is evidence from eukaryotic systems that protein *O*-acylation is reversible, which in principle allows for dynamic regulation of protein location or activity, as observed in other PTMs such as phosphorylation and *O*-GlcNAcylation. For example, deacylation of *O*-palmitoleoylated Wnt proteins, which is catalyzed by the carboxylesterase Notum, regulates Wnt-mediated signaling;^77^*S*-palmitoylation is also a reversible form of protein lipidation.^1^ Some of the proteins we identified here as *O*-mycoloylated, notably the well-characterized mycoloyltransferases and porins, are known to be found in the culture medium in their nonacylated forms. It is possible that under appropriate conditions, a secreted hydrolase(s) can cleave the *O*-mycoloyl group(s) from these and other *O*-mycoloylated proteins to regulate their location and/or function. To date, such an activity has not been discovered. As future studies continue to explore the occurrence and functional consequences of protein *O*-mycoloylation (and potentially de-*O*-mycoloylation), chemical tools such as those reported here will continue to play a key role.

## Conclusions

In summary, this study combined specific metabolic labeling, click chemistry, cleavable linker technology, engineered bacterial strains, and a custom LC-MS/MS method to provide the first comprehensive analysis of bacterial protein *O*-mycoloylome. In a significant expansion of the known breadth of this PTM, we identified approximately 30 proteins as *O*-mycoloylated in Cg, which is a model organism for pathogens of high medical significance in the *Corynebacterineae* suborder of bacteria. As anticipated, the known or predicted functions of these proteins mainly involve cell envelope biosynthesis, remodeling, or transport, which strongly supports the hypothesis that *O*-mycoloylation targets proteins to the mycomembrane and tethers them there, at least transiently, to perform their function. Notably, our strategy suggested for the first time the putative *O*-mycoloylation of mycoloyltransferases, which have been the subject of intensive study for decades due to their critical roles in bacterial physiology and pathogenesis and their corresponding attractiveness as targets for drug, diagnostic, and vaccine development. We also disclosed here the first proteome-wide *O*-mycoloylation site mapping data, which provide an unprecedented view of the nature of the PTM, namely that it occurs on flexible and uncongested peptide motifs and often in the form of multiple modifications in close spatial proximity to one another. One limitation of our strategy is that bottom-up chemical proteomics is not ideal for analyzing small *O*-mycoloylated proteins, such as Cg porins. In addition, the O-AlkTMM probe exhibited a minor amount of Cmt1-independent incorporation into proteins. Although this was ameliorated in the present study by including control experiments with mutant strains, there is interest in optimizing the specificity of TMM probes through structural refinement of the clickable acyl chain to more closely resemble native mycolates. Regardless, the tools reported here can be applied in the future to (i) facilitate follow-up structural and functional characterization of putative *O*-mycoloylated proteins of interest in Cg, particularly the mycoloyltransferases, (ii) explore the dynamics of protein *O*-mycoloylation in various environmental contexts, (iii) search for and characterize the *O*-mycoloylation PTM in related pathogenic bacteria, and (iv) develop assays to identify and characterize inhibitors of protein *O*-mycoloylation. In addition to furthering our understanding of the scope and nature of bacterial *O*-mycoloylation, the chemical proteomics strategies described herein may be more broadly useful in the analysis of other types of post-translational protein lipidation.

## Data Availability

Raw data are available in MassIVE repository (MSV000091337).

## Supporting Information Available

List of strains used in this study (Table S1); list of plasmids used in this study (Table S2); list of primers used in this study (Table S3); efficiency of protein extraction method (Figure S1); comparison of O-AlkTMM and alkyne carboxylic acids (Figure S2); comparison of proteins identified in this study and predicted mycomembrane proteins (Figure S3); examples of annotated MS spectra (Figure S4); AlphaFold structures of *O*-mycoloylated proteins (Figure S5); general procedures; construction of mutants; Cmt1-dependent protein labeling; concentration-dependent protein labeling; cell labeling and flow cytometry analysis; click chemistry-mediated affinity enrichment using Az-T-B; label-free quantitative LC-MS/MS analysis (LC-MS/MS study 1); PorB labeling; click chemistry-mediated affinity enrichment using Az-DADPS-B; LC-MS/MS analysis and PTM site localization (LC-MS/MS studies 2 and 3); Supporting references (PDF)
LC-MS/MS raw and curated data for LC-MS/MS studies 1–3 (Tables S4–S11) (XLSX)
Lists of ions observed in HCD and AI-ETD spectra in Figure 3E,F (Tables S12 and S13) (XLSX)
