# Supplementary material for: Chemical Proteomics Strategies for Analyzing Protein Lipidation Reveal the Bacterial O-Mycoloylome
Source: J Am Chem Soc. 2024 Apr 18;146(17):12138–54. doi: 10.1021/jacs.4c02278 (PMC11066868; doi:10.1021/jacs.4c02278)
Supplement: Supplementary file 1 — ja4c02278_si_001.pdf [file ja4c02278_si_001.pdf]

Supplementary Information (SI)

for

Chemical proteomics strategies for analyzing protein lipidation reveal the bacterial *O*-mycoloylome

Nicholas Banahene,[1,2] Trenton M. Peters-Clarke,[3,4,5] Kyle J. Biegas,[1,2] Evgenia Shishkova,[4,5] Elizabeth M. Hart,[6,7] Amelia C. McKitterick,[6,7] Nikolas H. Kambitsis,[1] Ulysses G. Johnson,[1,2] Thomas G. Bernhardt,[6,7] Joshua J. Coon,[3,4,5,8] and Benjamin M. Swarts[1,2,*]

[1]Department of Chemistry and Biochemistry, Central Michigan University, Mount Pleasant, MI, 48859, USA

[2]Biochemistry, Cell, and Molecular Biology Graduate Programs, Central Michigan University, Mount Pleasant, MI, 48859, USA

[3]Department of Chemistry, University of Wisconsin, Madison, WI 53562, USA

[4]Department of Biomolecular Chemistry, University of Wisconsin, Madison, WI 53562, USA

[5]National Center for Quantitative Biology of Complex Systems, University of Wisconsin, Madison, WI 53562, USA

[6]Department of Microbiology, Harvard Medical School, Boston, MA, 02115 USA

[7]Howard Hughes Medical Institute, Chevy Chase, MD 20815, USA

[8]Morgridge Institute for Research, Madison, WI 53562, USA

*Corresponding author: E-mail: ben.swarts@cmich.edu

SI Table of Contents*

Supplementary tables ____ S3
Table S1. List of strains used in this study ____ S3
Table S2. List of plasmids used in this study ____ S4
Table S3. List of primers used in this study ____ S5
Tables S4–S11. LC-MS/MS raw and curated data ____ *
Tables S12–S13. Figures 3E and 3F HCD and AI-ETD ion lists ____ **
Supplementary figures ____ S7
Figure S1. Efficiency of protein extraction method ____ S7
Figure S2. Comparison of O-AlkTMM and alkyne carboxylic acids ____ S8
Figure S3. Comparison of proteins identified in this study and predicted mycomembrane proteins ____ S8
Figure S4. Examples of annotated MS spectra ____ S9
Figure S5. AlphaFold structures of *O*-mycoloylated proteins ____ S10
Experimental Methods ____ S11
General procedures ____ S11
Construction of mutants ____ S13
Cmt1-dependent protein labeling ____ S14
Concentration-dependent protein labeling ____ S15
Cell labeling and flow cytometry analysis ____ S15
Click chemistry-mediated affinity enrichment using Az-TB ____ S16
Label-free quantitative LC-MS/MS analysis (LC-MS/MS Study 1) ____ S17
PorB labeling ____ S19
Click chemistry-mediated affinity enrichment using Az-DADPS-B ____ S19
LC-MS/MS analysis and PTM site localization (LC-MS/MS Studies 2 and 3) ____ S22
SI References ____ S24

***LC-MS/MS raw and curated data available separately as "Supplementary Tables S4-11_Proteomic Analysis.xlsx"**

****Lists of ions observed in HCD and AI-ETD spectra in Figures 3E and 3F available separately as "Supplementary Tables S12-13_HCD and AI-ETD Ions.xlsx"**

Table S1. List of strains used in this study

Strain number	Genotype	Strain construction	Source/ reference
H60	MB001 (ATCC 13032 ΔCGP1 (cg1507-gp1524) ΔCGP2 (cg1746-1752) ΔCGP3 (cg1890-cg2071))		[1]
H1664	Δ*cmt1* (Δ*cgp_0413)*	MB001/pACM20	[2]
H2265	Δ*cmt1 attB1::cmt1*	H1664/pACM64	[2]
H1119	Δ*porB* (*cgp_1109*)	MB001/pEMH2	This study
H1114	Δ*porB* pEMH25	H1119/pEMH25	This study
H1358	Δ*porB* pEMH61	H1119/pEMH61	This study
H1391	Δ*porB* pEMH86	H1119/pEMH86	This study

Table S2. List of plasmids used in this study.

Plasmid name	Information	Source/reference
pACM185	P_{sod} riboE1 empty vector (Kan^R, pK-PIM derivative, theophylline inducible)	[2]
pACM64	P_{sod} riboE1::*cmt1* (Kan^R, pK-PIM derivative, theophylline inducible)	[2]
pCRD206	Kan^R, *sacB* counterselection, temperature-sensitive origin	[3]
pACM20	pCRD206::*cmt1* (Kan^R, pCRD206 derivative containing an insert covering upstream and downstream of *cmt1*)	[2]
pEMH2	pCRD206::*porB* (Kan^R, pCRD206 derivative containing an insert covering upstream and downstream of *porB*)	This study
pTGR5	$P_{tac::}$*gfp* (Kan^R, P_{TAC}-eGFP, pGA1 mini replicon)	[4]
pEMH25	P_{sod} empty vector (Cam^R, pGA1 mini replicon, constitutive expression)	This study
pEMH61	P_{sod}::*porB-6xHis* (Cam^R, pGA1 mini replicon, constitutive expression)	This study
pEMH86	P_{sod}::*porB*$_{S7A/S98A}$*-6xHis* (Cam^R, pGA1 mini replicon, constitutive expression)	This study

Table S3. List of primers used in this study.

Primer number	Sequence (5' to 3')	Description (associated plasmid)
BH1	AGTCGACCTGCAGGCATG	Forward, to linearize pCRD206 backbone, (pEMH2)
BH2	ATCCAACAGGGACACCAG	Reverse, to linearize pCRD206 backbone, (pEMH2)
BH27	tcctggtgtccctgttggatATGATTGGAGGGGTTGCAAC	Forward, to amplify upstream region of *porB* (pEMH2)
BH175	tgaagaaggaAAGCTTCATGATTTTTAGGGCTC	Reverse, to amplify upstream region of *porB* (pEMH2)
BH52	tgcatgcctgcaggtcgactCGAATTCCTCGACTCTGATTC	Forward, to amplify downstream region of *porB* (pEMH2)
BH176	catgaagcttTCCTTCTTCACTGCTTAG	Reverse, to amplify downstream region of *porB* (pEMH2)
BH41	aagtccgccatgcccgaa	Forward, to amplify pTGR5 vector to remove *lacI*/P_{tac}/*eGFP* (pEMH25)
BH42	tgagcgcaacgcaattaatgtaag	Reverse, to amplify pTGR5 vector to remove *lacI*/P_{tac}/*eGFP* (pEMH25)
BH43	cattaattgcgttgcgctcaaagcgcctcat cagcggt	Forward, to amplify native P_{sod} promoter and RBS from MB001 genome (pEMH25)
BH44	ccttcgggcatggcggacttgggtaaaaaa tcctttcgtaggtttcc	Reverse, to amplify native P_{sod} promoter and RBS from MB001 genome (pEMH25)
J273	gcgggactctggggttcg	Forward, to amplify pTGR5 derivatives to remove kanamycin-resistance cassette (pEMH25)
J274	gcgaaacgatcctcatcctgtct	Reverse, to amplify pTGR5 derivatives to remove kanamycin-resistance cassette (pEMH25)
J275	agacaggatgaggatcgtttcgc atggagaaaaaaatcactggatataccacc	Forward, to amplify chloramphenicol-resistance cassette (pEMH25)

J276	cgaaccccagagtcccgcttacgccccgcc ctgcc	Reverse, to amplify chloramphenicol-resistance cassette (pEMH25)
BH148	tgacctaggtgcctggcg	Forward, to delete region downstream of P_{sod} promoter (pEMH25)
BH149	gggtaaaaaatcctttcgtaggtttcc	Reverse, to delete region downstream of P_{sod} promoter (pEMH25)
BH196	cttctcttcccaccaccaccaccaccac	Forward, to amplify pEMH25-derived 6x His backbone for insertion of *porB* (pEMH61)
BH197	aaagcttcatgggtaaaaaatcctttcgtaggtttcc	Reverse, to amplify pEMH25-derived 6x His backbone for insertion of *porB* (pEMH61)
BH198	ttttttacccatgaagctttcacaccgc	Forward, to amplify *porB* from MB001 genome (pEMH61)
BH199	ggtggtggtgggaagagaagttggaggac	Reverse, to amplify *porB* from MB001 genome (pEMH61)
BH251	tgcttccgcagcagacttcgcaaac	Forward, to mutate S7A on *porB* via site-directed mutagenesis (pEMH86)
BH252	ggtgctgcgaatgctgcc	Reverse, to mutate S7A on *porB* via site-directed mutagenesis (pEMH86)
BH253	ctccaacttcgcatcctaacctag	Forward, to mutate S98A on *porB* via site-directed mutagenesis (pEMH86)
BH254	gacagctcagaaaggtag	Reverse, to mutate S98A on *porB* via site-directed mutagenesis (pEMH86)

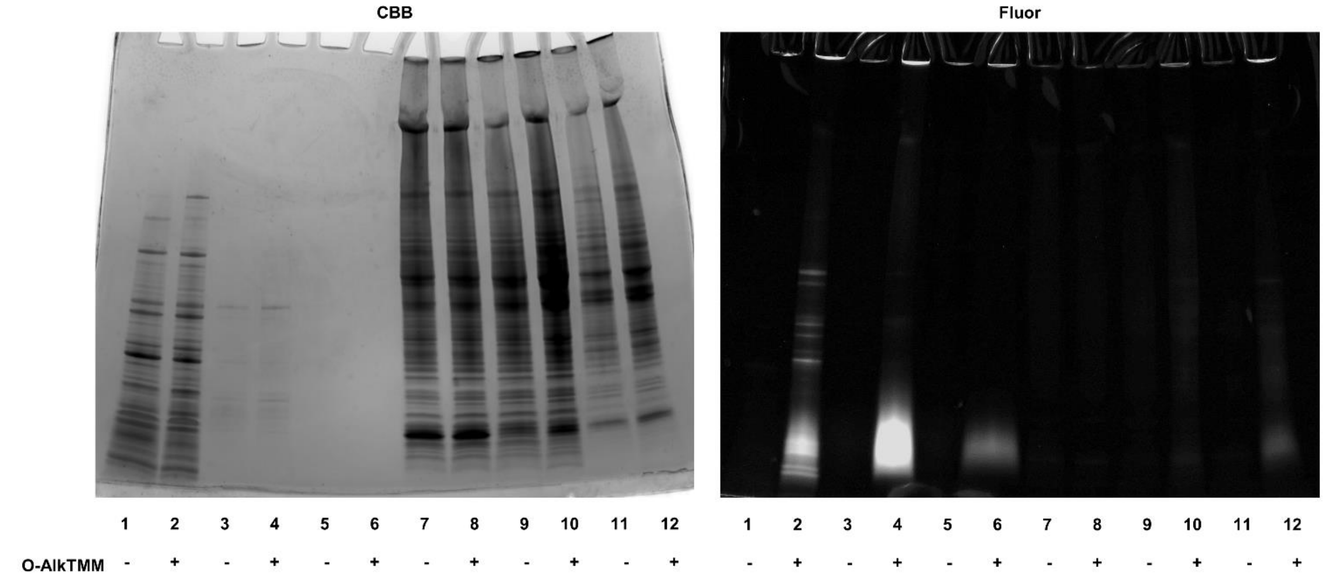

Supplementary Figure S1. Efficiency of protein extraction method. *Cg* wild type (WT) was cultured in the presence of 100 μM O-AlkTMM or left untreated for 16 h, then proteins were extracted successively three times, each by boiling at 100 °C in 4% SDS for 5 min (lanes 1-6: lanes 1&2, first extraction; lanes 3&4, second extraction; lanes 5&6, third extraction). The pellet remaining from these initial three extractions was subjected to three further successive extractions, each carried out by heating to 60 °C in PBS containing 2 mg/mL lysozyme for 2 h, bead beating, and extraction with 2% SDS at 60 °C for 2 h (lanes 7-12: lanes 7&8, fourth extraction; lanes 9&10, fifth extraction; lanes 11&12, sixth extraction). The proteins from each extraction step were subjected to CuAAC with azido-488, separated by SDS-PAGE, and visualized by Coomassie Brilliant Blue (CBB) staining and in-gel fluorescence scanning.

Supplementary discussion of Figure S1. Treatment of *Cg* cells with 4% SDS at 100 °C for 5 min was previously reported to extract cell envelope-associated proteins,[5-7] which we predicted would serve as a convenient and efficient method for extraction of labeled proteins. To test this method, we labeled *Cg* WT with O-AlkTMM, then performed three successive extractions under these conditions (4% SDS at 100 °C for 5 min), click-labeled proteins with azido-488, and analyzed the proteins. We found that fluorescently labeled, putatively *O*-mycoloylated proteins were mainly present in the first two rounds of extraction (lanes 1-4), with minimal total protein or fluorescence signal in the third extraction (lanes 5 and 6). To stringently test whether any labeled proteins remained, the pellets were subjected three times to treatment with lysozyme, bead beating, and extraction with 2% SDS at 60 °C for 2 h, and the protein extracts were processed and visualized as just described. We found that, while significant additional total protein was extracted from the remaining pellets, there was virtually no fluorescence in those proteins (lanes 7-12). Thus, 1-2 extractions of *Cg* cells using 4% SDS at 100 °C for 5 min is sufficient to efficiently extract all O-AlkTMM-labeled proteins, while also serving as a partial enrichment step for these proteins.

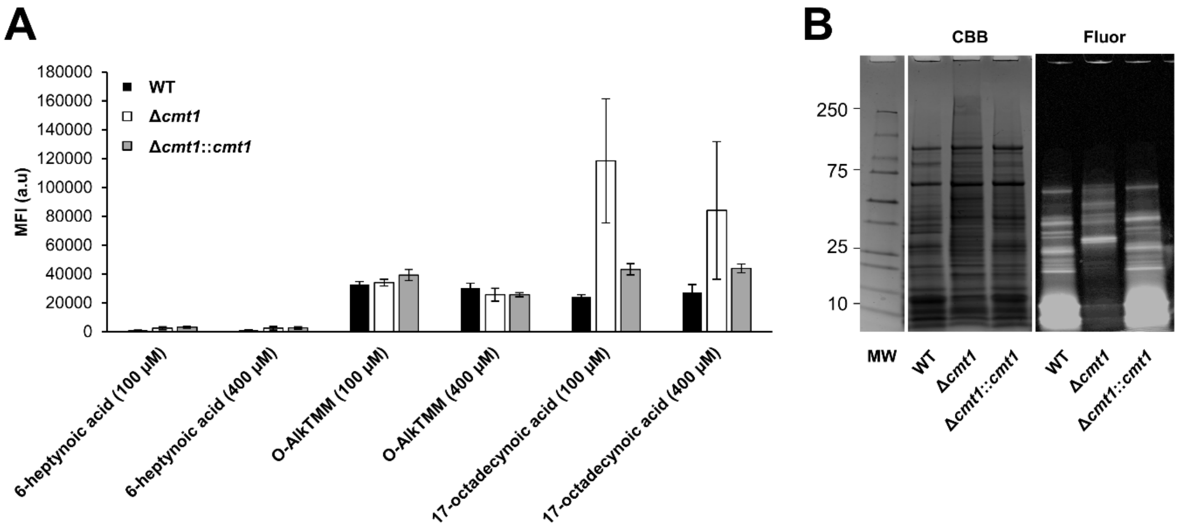

Supplementary Figure S2. Comparison of O-AlkTMM and alkyne carboxylic acids. (A) *Cg* wild type (WT), *cmt1* mutant (Δ*cmt1*), or complement (Δ*cmt1*::*cmt1*) were treated for 16 h with different concentrations of O-AlkTMM, 6-heptynoic acid, or 17-octadecynoic acid, or left untreated. Cells were fixed then, subjected to CuAAC with azido-488, and analyzed by flow cytometry. Error bars represent the standard deviation of three replicate experiments. MFI, mean fluorescence intensity; a.u., arbitrary units. (B) Cell lysates were collected from *Cg* strains treated with 400 µM 17-octadecynoic acid as in (A), subjected to CuAAC with azido-488, and analyzed by SDS-PAGE with visualization by Coomassie Brilliant Blue (CBB) staining and in-gel fluorescence scanning (Fluor).

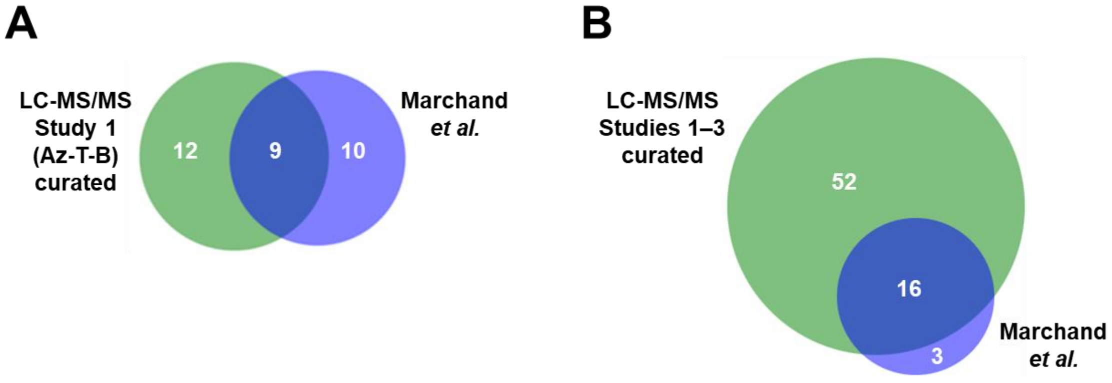

Supplementary Figure S3. Comparison of proteins identified in this study and predicted mycomembrane proteins. Venn diagram comparisons of proteins predicted to be mycomembrane-associated by Marchand *et al.*[8] and (A) the proteins identified in LC-MS/MS Study 1 (see Supplementary Table S6) and (B) the combined proteins identified in LC-MS/MS Studies 1–3 (Supplementary Tables S6 (Study 1), S8 (Study 2), and S10 (Study 3)).

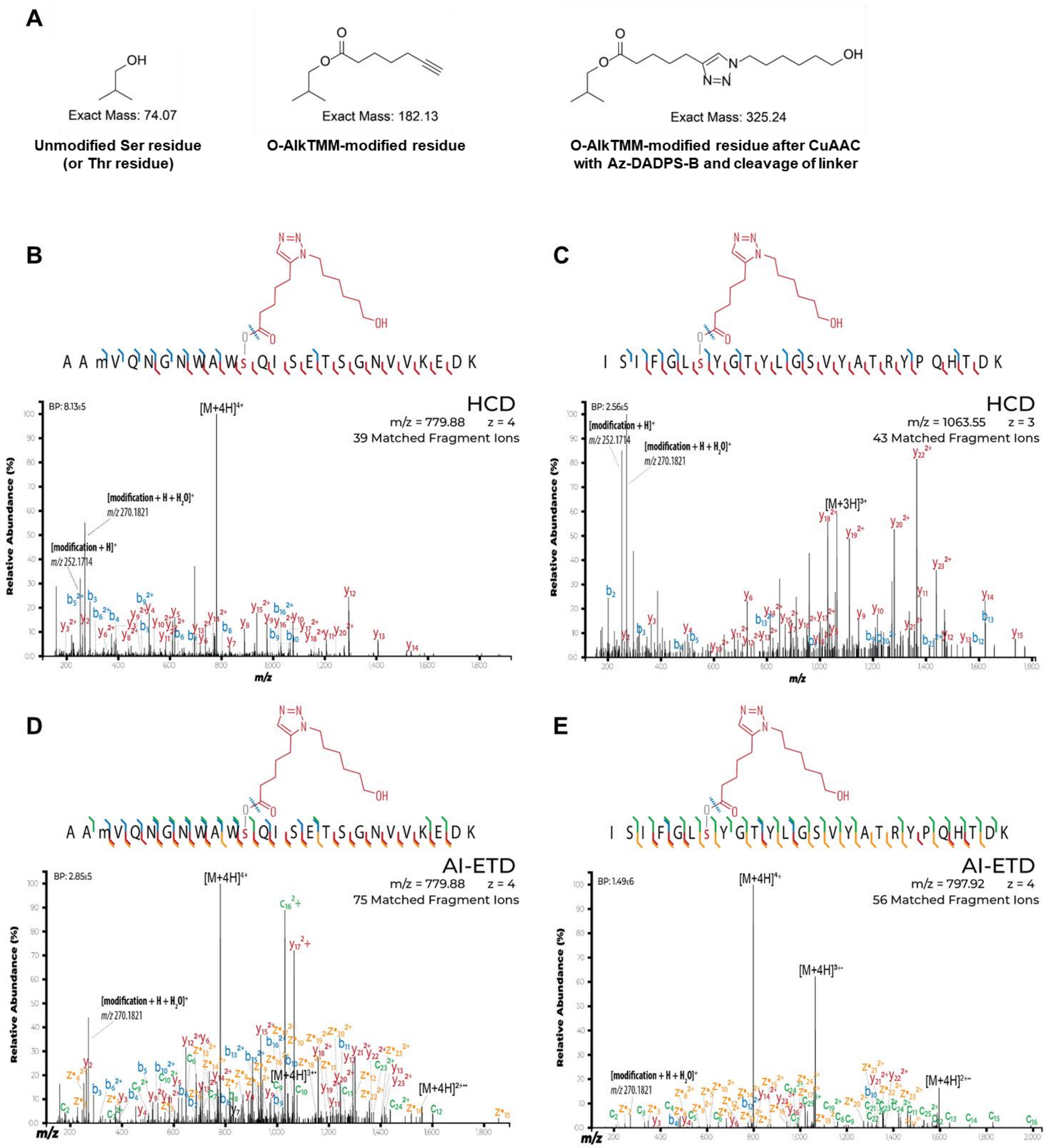

Supplementary Figure S4. Examples of annotated MS spectra. (A) Chemical structures of unmodified, O-AlkTMM-modified, and O-AlkTMM- and DADPS-modified and release peptides. (B-E) Examples of annotated (B and D) HCD and (C and E) AI-ETD spectra demonstrating thorough sequence coverage, confident site localization, and the presence of modification-related ions at m/z of 252.17 and 270.18.

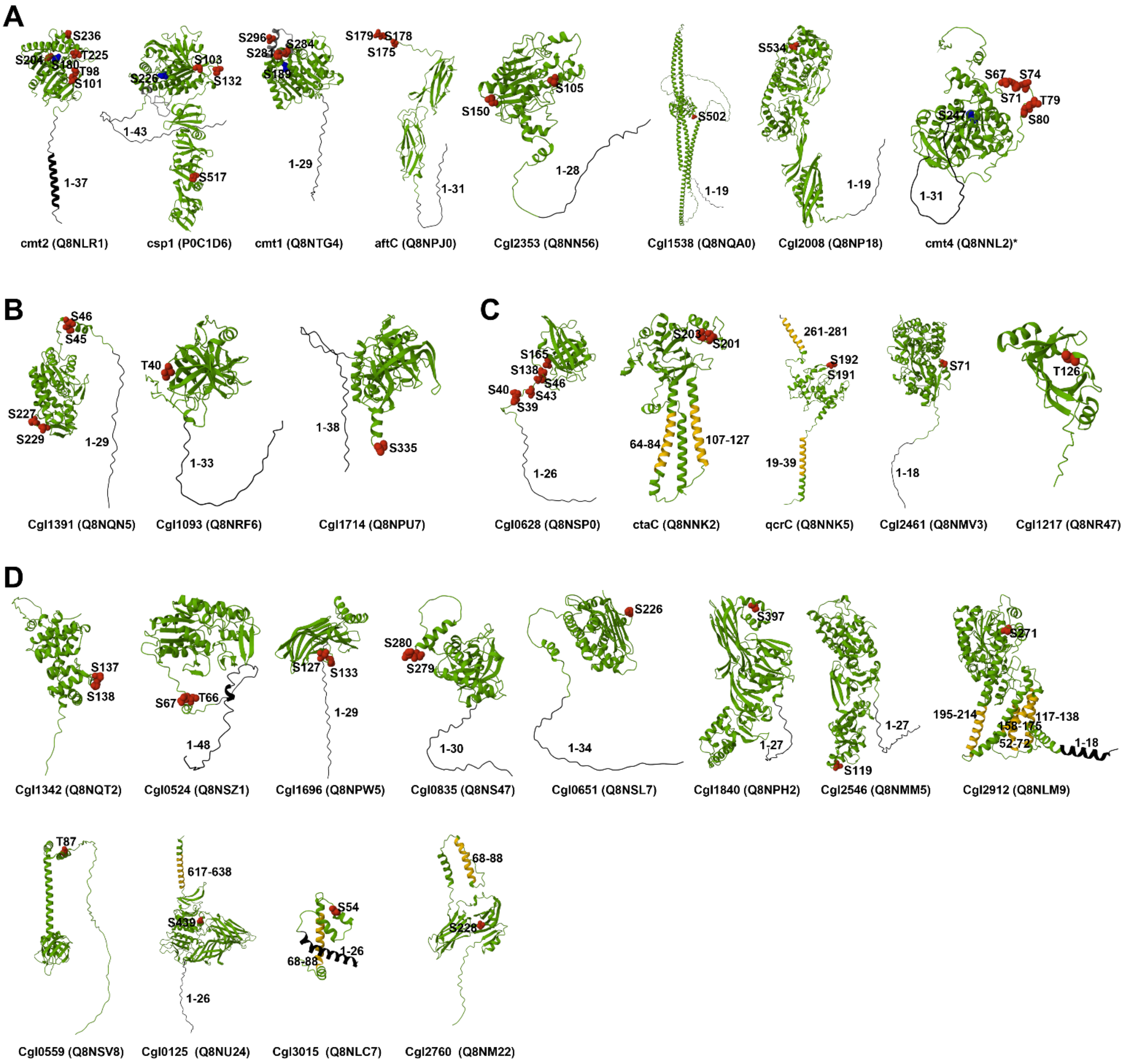

Supplementary Figure S5. AlphaFold structures of *O*-mycoloylated proteins. Shown are AlphaFold structures of proteins identified in Study 3 and organized as in Table 1: (A) Cell wall synthesis and remodeling proteins. (B) Hydrolases. (C) Other proteins. (D) Uncharacterized proteins. Gene names and UniProt IDs are given for each structure and residues of interest are annotated. Modification sites, red; catalytic residues for mycoloyltransferases in (A), blue; predicted signal sequences, black; predicted transmembrane domains, yellow. *In panel (A), the structure of Cmt4 is also shown although this protein was only identified in LC-MS/MS Study 2.

II. Experimental Methods

General procedures

Bacterial strains and growth conditions. *Corynebacterium glutamicum* (*Cg*) type strain 534 (ATCC 13032), Δ*cmt1* (clean deletion of *cmt1* in the wild-type strain 534 background, reported in ref.[9]), MB001 (ATCC13032 3xΔprophages), and MB001 Δ*cmt1* (clean deletion of *cmt1* in the MB001 background, construction reported herein) were cultured in LB media (pH 7.2). MB001 Δ*cmt1*::*cmt1* (*cmt1* complementation in the MB001 *Δcmt1* background, construction reported herein) was cultured in LB medium (pH 7.2) supplemented with 15 µg/mL kanamycin and 1 mM theophylline. MB001 Δ*porB* pTGR5(Psod/RBSsod) EV, MB001 Δ*porB* pTGR5(Psod/RBSsod)::*porB*-His and MB001 Δ*porB* pTGR5(Psod/RBSsod)::*porB*-S7A/S98A-His (construction of these strains is reported herein) were cultured in LB media (pH 7.2) supplemented with 3.5 µg/mL chloramphenicol. See Table S1 for additional information about strains constructed in this study.

Reagents. Stock solutions of O-AlkTMM (synthesized and characterized by NMR spectroscopy as in ref.[10]), 6-heptynoic acid (Sigma), and 17-octadecynoic (Cayman Chemical) acid were prepared in DMSO at concentrations of 100 mM, sterile filtered (0.45 µm), and stored at -20 °C. Other reagent stocks prepared for this study included: carboxyrhodamine 110 Azide (Az-488, Click Chemistry Tools, 1 mM in DMSO, stored at -20 ºC); DADPS Biotin Azide (Az-DADPS-B, Click Chemistry Tools, 10 mM in DMSO, stored at -20 ºC); TAMRA Biotin Azide (Az-T-B, Click Chemistry Tools, 10 mM in DMSO, stored at -20 ºC); sodium ascorbate (60 mM in Milli-Q water, freshly prepared); tris[(1-benzyl-1H1,2,3-triazol-4-yl)methyl]amine (TBTA) ligand (Click Chemistry Tools, 6.4 mM in *tert*-butanol/DMSO 3:1, stored at -20 ºC); copper(II) sulfate (50 mM in Milli-Q water, stored at 4 °C); sequence grade modified trypsin (Promega); lysyl endopeptidase (Lys-C, Fijufilm); 0.1 mm zirconia/silica beads (BioSpec Products); high-capacity

streptavidin agarose resin (Thermo Fisher Scientific); HisPur Ni-NTA resin (Thermo Fisher Scientific).

Protein labeling. *Cg* starter culture was diluted with LB liquid medium to an OD_{600} of 0.3 in a sterile flask. Next, alkyne probe was added to a desired final concentration, or DMSO control was added. The cell cultures were incubated for 16 h at 30 °C with constant shaking at 222 rpm, then harvested by centrifugation at 5,000 xg for 10 min at 20 °C and washed three times with phosphate-buffered saline (PBS).

***Protein extraction*.** Air-dried cell pellets containing alkyne-labeled proteins were suspended in 60 mM Tris-HCl at pH 6.8 containing 4% SDS and boiled at 100 °C for 5 mins. The supernatant, expected to contain cell wall-associated proteins, was collected by centrifugation at 13,000 xg for 15 mins at 4 °C. The extraction was repeated twice, and the clear supernatants were combined. This method was used for protein extraction except otherwise stated.

***Protein precipitation using chloroform/methanol/water*.** One part of the alkyne-labeled protein extract was added to four parts of ice-cold methanol and one part of ice-cold chloroform and vortexed briefly. Three parts of water were added, vortexed briefly, and centrifuged at 18,000 xg for 2-10 mins at 4°C. The top aqueous layer was carefully removed without disturbing the protein disc at the interface layer. Three parts of ice-cold methanol were added, vortexed briefly, and centrifuged at 18,000 xg for 2-10 mins at 4°C to pellet the proteins. The supernatant was discarded, and the precipitated proteins were air-dried briefly and resuspended in resuspension buffer (Milli-Q water containing 0.5% SDS and 0.05% LDAO).

***Copper-catalyzed azide–alkyne cycloaddition (CuAAC) conditions*.** To resuspended alkyne-labeled proteins were added in sequential order, azido reagent, sodium ascorbate (1.2 mM), TBTA (128 µM), and copper(II) sulfate (1 mM). The mixture was vortexed briefly and

incubated at 37 °C for 2 h.

SDS-PAGE analysis. Proteins were electrophoretically separated on a 4-20% polyacrylamide gel (Bio-Rad) in Tris-glycine-SDS running buffer, followed by fluorescence scanning using a Typhoon FLA 7000 (GE Healthcare Life Science) using either the FITC or rhodamine filter. The gel was fixed for 15 min (40% ethanol, 10% acetic acid in Milli-Q water), rinsed three times with Milli-Q water for 10 min, and stained overnight with gentle agitation in QC Colloidal Coomassie stain (Bio-Rad). The gel was rinsed with Milli-Q water until the background was clear, imaged using a ChemiDoc Touch Imaging System (Bio-Rad), and processed using Image Lab software version 6.1 (Bio-Rad). Silver staining was done using Pierce Silver Staining Kit (Thermo Fisher Scientific) and following the manufacturer protocol.

Construction of *Cg* mutant strains

Growth conditions. *C. glutamicum* strains were grown in brain heart infusion (BHI) medium (BD) at 30°C with aeration. Ectopic complementation constructs were induced with theophylline, as indicated. *E. coli* cloning strains were grown in LB medium (1% tryptone, 0.5% yeast extract, 0.5% NaCl) with aeration at 37°C. *E. coli* strains harboring pCRD206 derivatives were grown at 30°C with aeration. *C. glutamicum* strains were grown in 15μg/mL kanamycin (Kan) or 3.5μg/mL chloramphenicol (Cam) and *E. coli* strains were grown in 25μg/mL kanamycin or 25μg/mL chloramphenicol, when appropriate.

Plasmid construction. Plasmids were constructed using isothermal assembly (ITA) and were transformed directly into *E. coli* DH5α(λpir) competent cells. Primers used in plasmid construction are listed in Table S3. To construct pCRD206 derivatives, 500bp-1kb fragments encoding homology regions upstream and downstream of the gene of interest were amplified and assembled onto pCRD206[3] through ITA. The pEMH25 empty vector was constructed by first replacing *lacI*/P_{tac}/*eGFP* from the published pTGR5 vector[4] with the P_{sod} promoter native to

C. glutamicum (including the native RBS) through ITA. This kanamycin resistance cassette on the resulting was replaced with a chloramphenicol resistance cassette using ITA. Finally, Q5 mutagenesis was used to delete the region downstream of the P_{sod} promoter containing a 6x His tag. *porB* was amplified from MB001 genomic DNA and cloned onto a pEMH25 derivative containing a 6x His tagged construct via ITA. Site-directed mutagenesis of *porB-6xHis* was performed using the KLD mutagenesis mix (NEB), following manufacturer specifications. All plasmid constructs were confirmed by Sanger sequencing.

Strain construction. *C. glutamicum* competent cells were prepared as previously described.[11, 12] Gene deletion in *C. glutamicum* was performed using the temperature-sensitive plasmid pCRD206 with *sacB* counterselection.[3] Briefly, the pCRD206 derivative containing homology regions of the gene of interest was electroporated into wild-type MB001 *C. glutamicum*. To recover transformants, cells were plated to BHI + kanamycin at 24°C to allow for plasmid replication. Transformants were restreaked to BHI + kanamycin at 30°C to isolate plasmid integrants. Integrants were plated to BHI + 10% sucrose at 30°C to select against *sacB* encoded on the pCRD206 vector. Candidate mutants were further screened by replica patching to BHI and BHI + kanamycin to identify kanamycin-sensitive colonies. Deletion mutants were confirmed by performing colony PCR using primers that anneal upstream and downstream to the gene of interest. Integration of pK-PIM derivative plasmids[13] was validated by colony PCR.

Cmt1 dependence of protein labeling

10 mL cultures of *Cg* MB001, MB001 Δ*cmt1*, or MB001 Δ*cmt1*::*cmt1* in LB medium at an OD_{600} of 0.3 were treated with O-AlkTMM or 17-octadecynoic acid to a final concentration of 0.1 mM and incubated for 16 h. Protein extracts were collected, precipitated in chloroform/methanol/water, and resuspended in resuspension buffer. The resuspended proteins were reacted with Az-488 (20 µM) under CuAAC conditions, and excess unreacted reagents

were removed using chloroform/methanol/water precipitation. The proteins were air-dried briefly and dissolved in resuspension buffer, then equal amounts of each sample were resolved using SDS-PAGE and analyzed using in-gel fluorescence scanning and Coomassie staining.

Probe concentration dependence of protein labeling

10 mL cultures of *Cg* MB001 in LB medium at an OD_{600} of 0.3 were treated with either O-AlkTMM or 6-heptynoic acid to final concentrations of 0.05, 0.1, 0.2, and 0.4 mM, or DMSO control, and incubated for 16 h. Protein extracts were collected, precipitated in chloroform/methanol/water, and resuspended in resuspension buffer. The resuspended proteins were reacted with Az-488 (20 µM) under CuAAC conditions, and excess unreacted reagents were removed using chloroform/methanol/water precipitation. The proteins were air-dried briefly and dissolved in resuspension buffer, then equal amounts of each sample were resolved using SDS-PAGE and analyzed using in-gel fluorescence scanning and Coomassie staining.

Cell labeling and flow cytometry analysis

In a 96 well plate, 198 µL cultures of *Cg* MB001, MB001 Δ*cmt1*, or MB001 Δ*cmt1*::*cmt1* in LB medium at an OD_{600} of 0.3 were treated with O-AlkTMM, 6-heptynoic acid, or 17-octadecynoic acid at concentrations of 0.1 or 0.4 mM, or DMSO control, and incubated for 16 h. The cells were pelleted by centrifugation at 3,200 xg for 5 min, washed three times with PBS containing 0.5 mg/mL bovine serum albumin (BSA), and fixed in 4% formaldehyde in PBS for 10 min. The cell was pelleted and washed three times with PBS and resuspended in 138 µL of PBS. Next, the cells were treated with Az-488 (20 µM) under CuAAC conditions and incubated in the dark at room temperature for 30 min. The cells were pelleted and washed to remove excess unreacted reagents and resuspended in 200 µL of PBS. 5 µL of the resuspended cells were added to 200 µL of PBS and analyzed on a CytoFlex flow cytometer (Beckman Coulter). Mean fluorescence intensity (MFI) values were collected for 50,000 events at an event rate of 500-

1,000 events/sec. The experiments were performed in triplicate.

Click chemistry-mediated affinity enrichment using Az-T-B

Protein labeling and extraction. 500 mL cultures of *Cg* type strain 534 at an OD_{600} of 0.3 were treated with O-AlkTMM to a final concentration of 0.4 mM, or DMSO control, and incubated for 16 h at 30 °C (for LC-MS/MS Study 1, n=4 replicates for both probe-treated and control conditions). For the Az-T-B enrichment study, proteins were extracted through a two-step method of (i) chloroform/methanol extraction and (ii) bead beating in resuspension buffer, then extracted proteins from each step were combined for subsequent analysis as follows. After the O-AlkTMM treatment step, the *Cg* cells were harvested, washed, air-dried briefly, resuspended in 120 mL of chloroform/methanol (2:1), and stirred for 10 h at room temperature. The cells were centrifuged at 5,000 xg for 10 min at room temperature, and the supernatants were saved. Next, the cell pellets were resuspended in 120 mL of chloroform/methanol (1:2), stirred at room temperature for 10 h, and pelleted by centrifugation, then the resulting cell pellets and the supernatants were saved. The supernatants from both chloroform/methanol extractions were combined and concentrated to near dryness by rotary evaporation. Next, these proteins were precipitated by adding 40 mL of ether and keeping at -20 °C for 10 h. The precipitated proteins were pelleted by centrifugation, air-dried, and resuspended in 500 µL of Milli-Q water, and an equal amount of methanol was added. Then chloroform/methanol (2:1 v/v) was added to a total volume of 40 mL and the sample was kept at -20 °C overnight. The precipitated proteins were pelleted by centrifugation, air-dried, and dissolved in the resuspension buffer. Next, the cell pellet remaining following the chloroform/methanol extractions was resuspended in resuspension buffer and stirred at 60 °C for 1 h, then transferred to screw-cap vials containing 0.25 mL of 0.1 mm silica/zirconia beads and subjected to bead beating at 5.5 m/s for 20 s (repeated 3 times). The beads were allowed to settle then the supernatants were gently

transferred into a 15 mL tube and centrifuged at 3,900 rpm for 5 min at room temperature. Next, the supernatant was gently transferred into another 15 mL tube and centrifuged at 10,000 xg for 5 min at room temperature. The clear supernatants were collected and combined with the proteins obtained by chloroform/methanol extraction. Next, ~1.5 mg of the combined protein extract was treated with Az-T-B (80 µM) under CuAAC conditions. Excess unreacted reagents were removed by chloroform/methanol/water precipitation as described above. The proteins were dried at 37 °C for 10 min and dissolved in 1 mL resuspension buffer.

Protein-level enrichment. ~100 µg of the 1.5 mg labeled protein were loaded onto 30 µL of settled pre-cleaned streptavidin agarose beads and incubated in the dark for 2 h at room temperature with end-over-end rotation. The beads were centrifuged at 2,000 xg for 1 min, and the supernatants were discarded. Next, the beads were washed twice with 50 mM Tris, 150 mM NaCl, and 1% SDS in water, twice with 1% SDS in 8 M urea, and twice with PBS. The bound proteins were eluted from the beads by boiling in 40 µL of 2x sample loading buffer at 95 °C for 15 min, separated using SDS-PAGE, and input and output protein sampls were analyzed by in-gel fluorescence and silver staining. The remaining ~1.4 mg of Az-T-B labeled proteins were prepared for subsequent LC-MS/MS analysis. Proteins were enriched on 100 µL of streptavidin beads and eluted as described above, stacked in a polyacrylamide gel, and stained overnight with QC Colloidal Coomassie to prepare for in-gel digestion and LC-MS/MS analysis.

LC-MS/MS analysis of peptides generated from Az-T-B enrichment (LC-MS/MS Study 1)

Protein digestion and LC-MS/MS analysis. The above method for click chemistry-mediated affinity enrichment of O-AlkTMM-labeled proteins using Az-T-B was carried out. Gel bands were digested in-gel, according to Shevchenko *et al.*,[14] with modifications. Briefly, gel bands were washed with 100 mM ammonium bicarbonate and dehydrated using 100% acetonitrile (ACN). Sequencing grade modified trypsin was prepared to 0.01 µg/µL in 50 mM

ammonium bicarbonate, and ~100 µL of this was added to each gel band to submerge the gel completely. Bands were then incubated at 37 °C overnight. Peptides were extracted from the gel by water bath sonication in a 60% ACN/1% trifluoroacetic acid (TFA) solution and vacuum dried to ~2 µL. The samples were resuspended to 20 µL using 2% ACN/0.1% TFA. Next, 5 µL was injected automatically using a Thermo EASYnLC 1200 onto a Thermo Acclaim PepMap RSLC 0.1 mm x 20 mm C18 trapping column and washed for ~5 min with buffer A. Bound peptides were then eluted onto a Thermo Acclaim PepMap RSLC 0.075 mm x 500 mm C18 resolving column over 35 min with a gradient of 8% B to 40% B in 24 min, ramping to 90% B in 1 min and held at 90% B for the duration of the run at a constant flow rate of 300 nL/min. Buffer A = 99.9% water/0.1% formic acid, Buffer B = 80% ACN/0.1% formic acid/19.9% H_2O. The column's temperature was maintained at a constant temperature of 50 °C using an integrated column oven (PRSO-V2, Sonation GmbH, Biberach, Germany). Eluted peptides were sprayed into a ThermoScientific Q-Exactive HF-X mass spectrometer using a FlexSpray spray ion source. Survey scans were taken in the Orbitrap (60,000 resolution, determined at *m*/*z* 200). The top fifteen ions in each survey scan were subjected to automatic higher energy collision-induced dissociation (HCD) with fragment spectra acquired at 15,000 resolution.

***Data analysis*.** The resulting MS/MS spectra were converted to peak lists using MaxQuant, v1.6.3.4, and searched against a protein database containing all *Cg* sequences appended with common laboratory contaminants (downloaded 2018-06-12 from www.ncbi.nlm.nih.gov and www.thegpm.org, respectively) using the Andromeda search algorithm, a part of the MaxQuant environment. The search output was then analyzed using Scaffold Q+S, v4.10.0, to probabilistically validate protein identifications. Assignments validated using the Scaffold 1% FDR confidence filter are considered true. Mascot parameters for all databases were as follows: allow up to 2 missed tryptic sites; Fixed modification of

Carbamidomethyl Cysteine; variable modification of Oxidation of Methionine, Acetylation of Protein N-terminus; peptide tolerance of +/- 10 ppm; MS/MS tolerance of 0.02 Da; FDR calculated using randomized database search.

PorB labeling

10 mL cultures of *Cg* MB001 strains constitutively expressing PorB wild type, PorB S7A/S98A double mutant, or an empty vector control in LB medium at an OD_{600} of 0.3 were treated with 100 µM of O-AlkTMM or DMSO control and incubated for 16 h. Protein extracts were collected, precipitated in chloroform/methanol/water, and resuspended in resuspension buffer. The resuspended proteins were reacted with Az-488 (20 µM) under CuAAC conditions, and excess unreacted reagents were removed using chloroform/methanol/water precipitation. The proteins were air-dried briefly and dissolved in resuspension buffer, then 100 µg of the proteins were diluted (1:10) in equilibration buffer (20 mM sodium phosphate, 300 mM sodium chloride, 20 mM imidazole; pH 7.4), loaded onto 100 µL equilibrated Ni-NTA resin, and incubated for 2 h at room temperature with end-over-end rotation. The beads were pelleted by centrifugation at 700 xg for 2 min and washed five times for 5 min each with 1 mL wash buffer (20 mM sodium phosphate, 300 mM sodium chloride, 25 mM imidazole; pH 7.4). The bound proteins were eluted three times with 100 µL of elution buffer (20 mM sodium phosphate, 300 mM sodium chloride, 300 mM imidazole; pH 7.4). The combined supernatant was dried on a speed-vac, dissolved in 50 µL of 2x gel loading buffer, resolved using SDS-PAGE, and analyzed using in-gel fluorescence scanning and Coomassie staining.

Click chemistry-mediated affinity enrichment using Az-DADPS-B

Protein labeling and extraction. 500 mL cultures of *Cg* type strain 534 or the corresponding Δ*cmt1* mutant[9] at an OD_{600} of 0.3 were treated with O-AlkTMM to a final concentration of 0.4 mM, or DMSO control, and incubated for 16 h at 30 °C (for sample preparation for LC-MS/MS Study 2, only wild-type *Cg* was used and n=2 for both probe-treated

and control conditions; for sample preparation for LC-MS/MS Study 3, both wild-type and Δ*cmt1* were used and n=4 for wild-type/probe-treated condition and n=3 for all control conditions). As described in the general procedures, protein extracts were collected, precipitated in chloroform/methanol/water, and resuspended in resuspension buffer. The resuspended proteins were reacted with Az-DADPS-B (100 µM) under CuAAC conditions, and excess unreacted reagents were removed using chloroform/methanol/water precipitation. Biotinylated proteins were then subjected to procedures for protein-level enrichment (Study 2) or peptide-level enrichment (Study 3) as follows.

Protein-level enrichment (LC-MS/MS Study 2). ~1 mg of biotinylated protein extracts from probe-treated and untreated *Cg* wild type prepared as described above were added to 100 µL of settled pre-cleaned streptavidin beads and incubated for 2 h at room temperature with end-over-end rotation. The beads were centrifuged at 2,000 xg for 1 min, and then the supernatants were discarded. The beads were washed three times with 1 mL of 50 mM Tris, 150 mM NaCl and 1% SDS, three times with 1% SDS in 8 M urea, and two times with PBS. To release proteins and enable analysis by SDS-PAGE, the beads were resuspended in 100 µL of Milli-Q water containing 5% formic acid to cleave the DADPS linker and incubated at room temperature for 2 h. The beads were pelleted at 2,000 xg for 1 min, and the supernatant was saved. The 5% formic acid cleavage step was repeated once. The beads were washed once with resuspension buffer and combined with the saved supernatants from both 5% formic acid treatments. The proteins were precipitated in chloroform/methanol/water, and equal amounts were separated using SDS-PAGE and analyzed using silver staining following the manufacturer protocol. To enable global identification and site mapping analysis of captured proteins by LC-MS/MS, they were subjected on-bead digestion followed by formic acid-mediated release of modified peptides as follows. After subjecting ~1.5 mg biotinylated proteins to capture on streptavidin beads as described

above, the beads were washed three times with 1 mL of 50 mM Tris, 150 mM NaCl and 1% SDS, three times with 1% SDS in 8 M urea, two times with PBS, and resuspended in 500 µL of 100 mM Tris containing 8 M urea. Next, tris(2-carboxyethyl)phosphine (TCEP) and chloroacetamide were added to final concentrations of 10 mM and 40 mM, respectively, and the beads were incubated at room temperature for 10 min. 40 µg of mass spectrometry-grade lysyl endopepdtidase were added and incubated for 4 h at room temperature. 100 mM Tris was added to bring the urea concentration to 2 mM, then 40 µg of sequencing grade modified trypsin was added and the beads were incubated overnight at room temperature with end-over-end rotation. The beads were washed three times with PBS and three times with Milli-Q water and combined to form presumed unmodified peptides. The modified peptides were released from the beads with two sequential treatments of 200 µL of 5% formic acid. Next, the beads were washed once with 400 µL of 50% ACN/water and 1% formic acid and the wash was combined with the formic acid-released samples to generate modified peptide samples. The peptide samples were desalted using a 10 mg Strata X reversed-phase solid phase extraction cartridge, concentrated on a speed-vac, and analyzed by LC-MS/MS as described below.

***Peptide-level enrichment (LC-MS/MS Study 3)*.** ~1.5 mg of biotinylated protein extracts from probe-treated and untreated *Cg* wild type or Δ*cmt1* mutant prepared as described above were dissolved in 500 µL of 8 M urea in 100 mM Tris. TCEP and chloroacetamide were added to final concentrations of 10 mM and 40 mM, respectively, and incubated at room temperature for 10 min. 100 mM Tris was added to bring the urea concentration to 2 mM, and 40 µg of sequence-grade modified trypsin was added and incubated overnight at RT with end-over-end rotation. Next, 40 µg of sequencing grade modified trypsin was added and incubated at room temperature for 4 h. The suspension was diluted with PBS to bring the urea concentration to 0.4 mM. Next, 100 µL of settled pre-cleaned streptavidin beads suspended in 200 µL of PBS were

added and incubated for 4 h at room temperature with end-over-end rotation. The beads containing bound, modified peptides were pelleted by centrifugation at 2,000 xg for 3 min, and the supernatant was discarded. The beads were washed three times with PBS and three times with Milli-Q water. The enriched modified peptides were released from the beads with two sequential treatments of 200 µL of 5% formic acid. Next, the beads were washed once with 400 µL of 50% ACN/water and 1% formic acid and the wash was combined with the formic acid-released samples to generate modified peptide samples. The modified peptide samples were desalted using a 10 mg Strata X reversed-phase solid phase extraction cartridge, concentrated on a speed-vac, and analyzed by LC-MS/MS as described below.

LC-MS/MS analysis of modified peptides generated from Az-DADPS-B enrichment (LC-MS/MS Studies 2 and 3)

LC-MS/MS analysis. These MS experiments were performed on an Orbitrap Fusion Lumos Tribrid mass spectrometer (ThermoFisher Scientific) coupled to a nanoflow Ultimate 3000 Dionex pump (ThermoFisher Scientific). The mass spectrometer was retrofitted to include a Firestar ti60 Synrad 60 W CO_2 continuous wave infrared (IR) laser (10.6 µm) (Mukilteo, WA) to allow for excitation of precursor ions within the quadrupole linear ion trap (*i.e.*, activated ion-electron transfer dissociation (AI-ETD)).[15] Modified peptide samples prepared as described above were separated using 120 min gradients on a 40 cm column packed in-house with C18 particles (BEH C18 1.7 µm, Waters) to 30,000 psi[16] held at 50 °C using a column heater. Mobile phase A (MPA) was 0.2% formic acid in H_2O, mobile phase B (MPB) was 0.2% formic acid in 90:10 isopropanol:acetonitrile with 5 mM ammonium formate and 120 min methods ramped from 0-2% MPB over 5 min at 380 nL/min, 2-32% MPB over 67 min at 280 nL/min, 32-70% MPB over 8 min at 260 nL/min, 70-82% MPB over 5 min at 225 nL/min, 82-87% MPB over 12 min at 225 nL/min, 87-95% MPB over 1 min at 225 nL/min, 95-100% MPB over 8 min at 225 nL/min, held

at 100% MPB for 3 min at 225 nL/min, 100-0% MPB over 1 min at 225 nL/min, and equilibrated at 0% MPB for 10 min at 225 nL/min.[17] ~500 ng peptides were injected per experiment. The spray voltage was +2.0 kV with respect to the ground. High-resolution MS^1 and MS^2 scans were acquired in the positive ion mode in the Orbitrap at 60,000 and 15,000 resolution, respectively. MS^1 scans were collected every 2 seconds with *m/z* range of 300-1,650, AGC target 1e6, and a maximum injection time of 50 ms. Peaks were selected for MS/MS in a data-dependent manner, including precursors with charge states 2-6 and dynamic exclusion set to 15 sec. MS^2 scans were collected with *m/z* range of 150-1,650, AGC target 5e4, and a maximum injection time of 150 ms. For ion activation, either higher-energy C-trap dissociation (HCD) or activated ion-electron transfer dissociation (AI-ETD) was performed. Each method was performed in technical (injection) duplicates for each sample. HCD was performed at 28 NCE, and AI-ETD was performed with the laser set to 12% maximum power (~10 W).

Data analysis**.** All files were searched using MSFragger (v. 3.5)[18] via FragPipe interface (v. 18.0). Spectra were searched against *Corynebacterium glutamicum* database downloaded from UniProt 2020101 and appended with a reverse decoy and common contaminant identifications. Default settings were used unless specifically stated. The custom variable modification with mass 251.1634 on S and T residues was added. Protease was set to "trypsin," and digest length was 6-65. PTMProphet[19] was enabled to localize ST:251.1634 mod with min probability of 0.5. MaxLFQ quantification was enabled via IonQuant[20] with default settings, match-between-runs enabled, and MaxLFQ min ions set to 1. IPSA webtool[21] was used to annotate spectra and produce figures. A FragPipe search was also performed that allowed for the modification to occur on any residue other than Ser/Thr but there were no peptide matches.

SI References

1. Baumgart, M.; Unthan, S.; Rückert, C.; Sivalingam, J.; Grünberger, A.; Kalinowski, J.; Bott, M.; Noack, S.; Frunzke, J., Construction of a prophage-free variant of Corynebacterium glutamicum ATCC 13032 for use as a platform strain for basic research and industrial biotechnology. *Appl Environ Microbiol* **2013,** *79* (19), 6006.
2. McKitterick, A. C.; Bernhardt, T. G., Phage resistance profiling identifies new genes required for biogenesis and modification of the corynebacterial cell envelope. *Elife* **2022,** *11*, e79981.
3. Okibe, N.; Suzuki, N.; Inui, M.; Yukawa, H., Efficient markerless gene replacement in Corynebacterium glutamicum using a new temperature-sensitive plasmid. *J Microbiol Methods* **2011,** *85* (2), 155.
4. Ravasi, P.; Peiru, S.; Gramajo, H.; Menzella, H. G., Design and testing of a synthetic biology framework for genetic engineering of Corynebacterium glutamicum. *Microb Cell Fact* **2012,** *11*, 147.
5. Peyret, J. L.; Bayan, N.; Joliff, G.; Gulik-Krzywicki, T.; Mathieu, L.; Schechter, E.; Leblon, G., Characterization of the cspB gene encoding PS2, an ordered surface-layer protein in Corynebacterium glutamicum. *Mol Microbiol* **1993,** *9* (1), 97.
6. Chami, M.; Bayan, N.; Peyret, J. L.; Gulik-Krzywicki, T.; Leblon, G.; Shechter, E., The S-layer protein of Corynebacterium glutamicum is anchored to the cell wall by its C-terminal hydrophobic domain. *Mol Microbiol* **1997,** *23* (3), 483.
7. Brand, S.; Niehaus, K.; Pühler, A.; Kalinowski, J., Identification and functional analysis of six mycolyltransferase genes of Corynebacterium glutamicum ATCC 13032: the genes cop1, cmt1, and cmt2 can replace each other in the synthesis of trehalose dicorynomycolate, a component of the mycolic acid layer of the cell envelope. *Arch Microbiol* **2003,** *180* (1), 33.
8. Marchand, C. H.; Salmeron, C.; Bou Raad, R.; Méniche, X.; Chami, M.; Masi, M.; Blanot, D.; Daffé, M.; Tropis, M.; Huc, E.; Le Maréchal, P.; Decottignies, P.; Bayan, N., Biochemical Disclosure of the Mycolate Outer Membrane of Corynebacterium glutamicum. *J Bacterio.* **2011,** *194* (3), 587.

9. Fiolek, T. J.; Banahene, N.; Kavunja, H. W.; Holmes, N. J.; Rylski, A. K.; Pohane, A. A.; Siegrist, M. S.; Swarts, B. M., Engineering the Mycomembrane of Live Mycobacteria with an Expanded Set of Trehalose Monomycolate Analogues. *ChemBioChem* **2019,** *20* (10), 1282.

10. Foley, H. N.; Stewart, J. A.; Kavunja, H. W.; Rundell, S. R.; Swarts, B. M., Bioorthogonal chemical reporters for selective in situ probing of mycomembrane components in mycobacteria. *Angew. Chem. Int. Edit.* **2016,** *55* (6), 2053.

11. Lim, H. C.; Sher, J. W.; Rodriguez-Rivera, F. P.; Fumeaux, C.; Bertozzi, C. R.; Bernhardt, T. G., Identification of new components of the RipC-FtsEX cell separation pathway of Corynebacterineae. *PLoS Genet* **2019,** *15* (8), e1008284.

12. van der Rest, M. E.; Lange, C.; Molenaar, D., A heat shock following electroporation induces highly efficient transformation of Corynebacterium glutamicum with xenogeneic plasmid DNA. *Appl Microbiol Biotechnol* **1999,** *52* (4), 541-545.

13. Oram, M.; Woolston, J. E.; Jacobson, A. D.; Holmes, R. K.; Oram, D. M., Bacteriophage-based vectors for site-specific insertion of DNA in the chromosome of Corynebacteria. *Gene* **2007,** *391* (1-2), 53.

14. Shevchenko, A.; Tomas, H.; Havlis, J.; Olsen, J. V.; Mann, M., In-gel digestion for mass spectrometric characterization of proteins and proteomes. *Nat. Protoc.* **2006,** *1* (6), 2856.

15. Peters-Clarke, T. M.; Schauer, K. L.; Riley, N. M.; Lodge, J. M.; Westphall, M. S.; Coon, J. J., Optical Fiber-Enabled Photoactivation of Peptides and Proteins. *Anal Chem* **2020,** *92* (18), 12363.

16. Shishkova, E.; Hebert, A. S.; Westphall, M. S.; Coon, J. J., Ultra-High Pressure (>30,000 psi) Packing of Capillary Columns Enhancing Depth of Shotgun Proteomic Analyses. *Anal Chem* **2018,** *90* (19), 11503.

17. He, Y.; Rashan, E. H.; Linke, V.; Shishkova, E.; Hebert, A. S.; Jochem, A.; Westphall, M. S.; Pagliarini, D. J.; Overmyer, K. A.; Coon, J. J., Multi-Omic Single-Shot Technology for Integrated Proteome and Lipidome Analysis. *Anal Chem* **2021,** *93* (9), 4217.

18. Kong, A. T.; Leprevost, F. V.; Avtonomov, D. M.; Mellacheruvu, D.; Nesvizhskii, A. I., MSFragger: ultrafast and comprehensive peptide identification in mass spectrometry-based proteomics. *Nat Methods* **2017,** *14* (5), 513.

19. da Veiga Leprevost, F.; Haynes, S. E.; Avtonomov, D. M.; Chang, H. Y.; Shanmugam, A. K.; Mellacheruvu, D.; Kong, A. T.; Nesvizhskii, A. I., Philosopher: a versatile toolkit for shotgun proteomics data analysis. *Nat Methods* **2020,** *17* (9), 869.

20. Yu, F.; Haynes, S. E.; Nesvizhskii, A. I., IonQuant Enables Accurate and Sensitive Label-Free Quantification With FDR-Controlled Match-Between-Runs. *Mol Cell Proteomics* **2021,** *20*, 100077.

21. Brademan, D. R.; Riley, N. M.; Kwiecien, N. W.; Coon, J. J., Interactive Peptide Spectral Annotator: A Versatile Web-based Tool for Proteomic Applications. *Mol Cell Proteomics* **2019,** *18* (8 suppl 1), S193.
